# Cell signaling and transcriptional regulation of osteoblast lineage commitment, differentiation, bone formation, and homeostasis

**DOI:** 10.1038/s41421-024-00689-6

**Published:** 2024-07-02

**Authors:** Siyu Zhu, Wei Chen, Alasdair Masson, Yi-Ping Li

**Affiliations:** Division in Cellular and Molecular Medicine, Department of Pathology and Laboratory Medicine, Tulane University School of Medicine, Tulane University, New Orleans, LA USA

**Keywords:** Developmental biology, Cell signalling, Mechanisms of disease

## Abstract

The initiation of osteogenesis primarily occurs as mesenchymal stem cells undergo differentiation into osteoblasts. This differentiation process plays a crucial role in bone formation and homeostasis and is regulated by two intricate processes: cell signal transduction and transcriptional gene expression. Various essential cell signaling pathways, including Wnt, BMP, TGF-β, Hedgehog, PTH, FGF, Ephrin, Notch, Hippo, and Piezo1/2, play a critical role in facilitating osteoblast differentiation, bone formation, and bone homeostasis. Key transcriptional factors in this differentiation process include Runx2, Cbfβ, Runx1, Osterix, ATF4, SATB2, and TAZ/YAP. Furthermore, a diverse array of epigenetic factors also plays critical roles in osteoblast differentiation, bone formation, and homeostasis at the transcriptional level. This review provides an overview of the latest developments and current comprehension concerning the pathways of cell signaling, regulation of hormones, and transcriptional regulation of genes involved in the commitment and differentiation of osteoblast lineage, as well as in bone formation and maintenance of homeostasis. The paper also reviews epigenetic regulation of osteoblast differentiation via mechanisms, such as histone and DNA modifications. Additionally, we summarize the latest developments in osteoblast biology spurred by recent advancements in various modern technologies and bioinformatics. By synthesizing these insights into a comprehensive understanding of osteoblast differentiation, this review provides further clarification of the mechanisms underlying osteoblast lineage commitment, differentiation, and bone formation, and highlights potential new therapeutic applications for the treatment of bone diseases.

## Introduction

Adult bone comprises three main cell types: osteoblasts, osteocytes (both of which are derived from mesenchymal stem cells (MSCs)), and osteoclasts (which are derived from hematopoietic stem cells (HSCs)). Regarding bone homeostasis, osteoblasts function to form bone tissue, osteoclasts resorb and degrade bone tissue, while the primary role of osteocytes is to maintain the structural integrity and function of bone by controlling the activity of both osteoclasts and osteoblasts. These opposing cell types regulate the physiological bone turnover rate, which can be separated into two stages: modeling and remodeling. Bone modeling happens continuously during development, while remodeling is involved in tissue renewal over the course of a person’s life. Bone remodeling typically initiates with osteoclasts breaking down the matrix, consisting mainly of type I collagen and crystalline hydroxyapatite proteins. Once resorption occurs, osteoblasts are recruited to the area to produce and mineralize fresh matrix. In the bone matrix, osteoblasts create a critical assortment of macromolecules, including collagen and non-collagen proteins, which act as a platform for lattice mineralization by means of hydroxyapatite formation. Imbalances in the quantity and ratio of osteoblasts to osteoclasts, stemming from bone tissue remodeling, can give rise to conditions such as osteoporosis and other phenotypically akin disorders. As such, there has been continued research on osteoblast differentiation to elucidate mechanisms with therapeutic potential. This review examines the recent advancements in the understanding of cell signaling, transcription, epigenetics, extracellular interplay, and physiological mechanisms that underpin osteoblast differentiation and their application to the treatment of related diseases.

## Osteoblast function

Osteoblasts, a major cellular component of bone and the terminal differentiation products of MSCs, have three main functions. First, osteoblasts play a pivotal role as the primary cellular regulator of bone formation. This involves synthesizing and releasing various proteins found in the extracellular matrix (ECM) of bone, along with orchestrating gene expression to facilitate ECM mineralization. With the assistance of β1 integrin, osteoblasts interface with the current matrix to shape cadherin-connected monolayers. Whenever osteoblasts are activated, they secrete a matrix consisting primarily of collagen type I and other vital components (Table 1), as well as significant developmental factors, such as bone morphogenetic protein (BMP) and transforming growth factor-β (TGF-β)^1^. Previous research also indicates that osteoblasts play important roles in glucose homeostasis via insulin signaling because it increases osteocalcin activity^2^.

**Table 1:** Osteoblast dysfunction-associated genes and their mouse models.

Gene	Role of gene/protein	Defective phenotype caused by mutation	Diseases caused by mutation	References
**AP1**	A transcriptional factor that can regulate osteoblast-specific genes, such as ALP, collagen type I, or OPN.	Osteopetrosis.	Embryonic lethality, persistent truncus arteriosus, congenital chronic diarrhea	^433,434^
**ATF4**	Determines the initiation and terminal differentiation of osteoblasts.	Osteoblast differentiation delay.	Coffin-Lowry Syndrome	^354^
**β-catenin**	Essential for preventing osteoblast transdifferentiation into chondrocytes or adipocytes and for osteoblast differentiation.	Inhibits osteoblast differentiation and instead develops into chondrocytes.	Cancer; Aggressive fibromatosis; Pulmonary fibrosis; Cleft palate; hyperparathyroidism-jaw tumor syndrome (HPT-JT)	^65,66,435–438^
**C/EBPβ and –δ**	A vital transcription factor. Runx2 can regulate C/EBP expression, and C/EBP induces the expression of OCN by direct binding within its promoter. It is necessary for osteoblast mineralization.	Deferred bone arrangement, obstructed osteoblast mineralization, and vital gene expression.	N/A	^439–445^
**CBFβ**	Through its interaction with transcriptional factors like Runxs and ATF4, it is an important factor in the formation of the skeleton.	Impairs osteoblast differentiation.	Skeletal deformities, CCD	^379–381^
**DLX3**	Positively and negatively regulates osteoprogenitor cell differentiation and gene transcription.	Inhibits induction of osteogenic markers.	Osteoporosis, Tricho-Dento-Osseous syndrome (TDO)	^446–449^
**DLX5**	Hinders adipocyte arrangement and manages the expression of transcription factors that control osteoblast differentiation.	Extreme craniofacial, pivotal, and affixed skeletal irregularities.	Split-hand/split-foot malformation (SHFM)	^450–454^
**ETS1**	Regulates OPN and ALP expression by cooperating with Runx2.	Runx2 and OPN were among the key genes whose expression was reduced, as was osteoblast differentiation.	N/A	^455–457^
**HES1**	A downstream effector of the Notch signaling pathway. Binds to Cbfα1 and potentiates its transactivating capability.	Defective functions of mammalian Runt-related proteins.	Severe neurulation defects	^458,459^
**HEY1**	A negative regulator of osteoblast differentiation and maturation.	Enhanced osteoblast matrix mineralization.	Potentially related to cardiac hypertrophy	^460–462^
**LEF1**	Regulates osteoblast differentiation by Wnt3a and BMP2 through binding with Runx2.	Affects osteoblast differentiation.	Human sebaceous adenoma, human sebaceoma	^375,463^
**MENIN**	Regulates osteoblast generation and differentiation, which are aided and sustained by TGF-β and BMP2.	Reduced ALP activation and OCN and Runx2 expression.	Human MEN1 disease, ossifying fibroma (OF), osteoporosis	^464–468^
**MSX2**	Inhibits adipocyte formation and stimulates mesenchymal cells to differentiate into osteoblasts.	Premature fusion of calvarial sutures.	Boston-type craniosynostosis	^469–471^
**Osterix**	Contributes to the final phase of osteogenesis and maturation, regulating the development of functional osteoblasts and their subsequent differentiation.	Devoid of osteoblasts.	Osteosarcoma; bone metastasis of cancers; Juvenile Paget’s disease (JPD)	^472^
**PPAR-γ**	Regulator of adipocyte differentiation and inhibits Runx2-mediated OCN transcription and osteoblast late differentiation.	High bone mass with increased osteoblastogenesis.	Partial lipodystrophy, insulin resistance, metabolic syndrome	^473–475^
**RUNX2**	Serves as the main switch for triggering the differentiation of osteoblasts and negatively regulates bone formation at the end of differentiation.	There are no osteoblasts, and the final stage of chondrocyte differentiation is blocked.	Delayed tooth eruption, cleidocranial dysplasia	^289,290^
**RUNX1**	A key regulator of osteoblast and chondrocyte homeostasis, it positively regulates osteoblast differentiation and inhibits adipocyte formation.	Reduced osteoblast number and cartilage were absent.	Osteoporosis	^311,315,317^
**RUNX3**	Participates in intramembranous and endochondral bone ossification during skeleton development.	The number of osteoblasts and mineral deposition capacity decreased.	Congenital osteopenia	^324,476^
**SALL4**	Promotes osteoblast differentiation by suppressing Notch2-targeted gene expression and nuclear translocation.	Osteoblast differentiation is inhibited.	Holt-Oram syndrome (HOS)	^238,477^
**SMAD**	Regulates targeted gene expression via interacting with Runx2, AP-1, and SP-1.	A phenotype similar to that of CCD.	CCD	^478^
**SMAD3**	Inhibits osteoblast differentiation and reduces osteocalcin and Cbfa1 expression.	Osteoblast differentiation rates declined.	Metastatic colon cancer	^114,479^
**STAT1**	Binding to Runx2 inhibits nuclear translocation of Runx2 and reduces Runx2 activity.	Excessive osteoblast differentiation and increased bone mass.	Be involved in human achondroplasia	^480–482^
**SATB2**	A molecular node controls osteoblast differentiation in the transcriptional network.	Osteoblast differentiation is lacking.	Cleft palate; SATB2-associated syndrome (SAS, Glass syndrome)	^483^
**TAZ/YAP**	Encourages the periosteal osteoblast precursors’ expansion and differentiation and acts with R-Smads to stimulate osteoblast-specific gene expression.	The number of differentiated osteoblasts decreased.	Skeletal fragility, long bone fractures	^484–487^

**Fig. 1:**
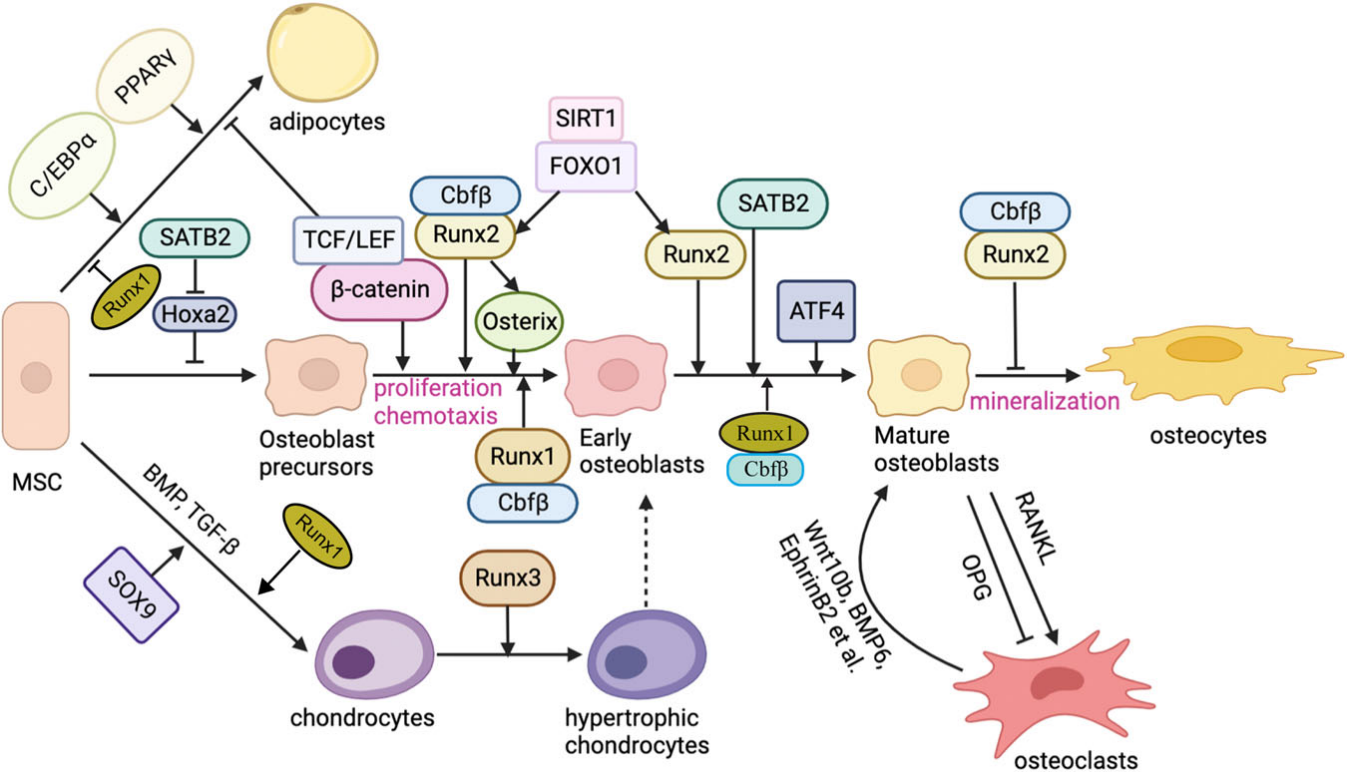
Different transcription factors regulate three different differentiation fates — adipocytes, osteoblasts, and chondrocytes from MSCs. MSCs have three different differentiation fates — adipocytes, osteoblasts, and chondrocytes — which are regulated by different genes. In the differentiation process, some cells in the three differentiation pathways also have a reciprocal transformation relationship through the regulation of related genes, such as interactions between mature osteoblasts and mature osteoclasts or hypertrophic chondrocytes and early osteoblasts. Transcription factors have different functions in different stages of osteoblast differentiation. Runx2 is a vital factor in all osteoblast differentiation stages; Runx2 promotes osteoblast differentiation in the early stage while inhibiting mature osteoblast differentiation into osteocytes. Cbfβ is the major co-factor of Runx2 and Runx1. Runx3 can promote chondrocytes into hypertrophic chondrocytes. SIRT1 and FOXO1 can promote Runx2 expression. Osx and β-catenin also have important functions in the early stage of osteoblast differentiation. SATB2 and ATF4 are important in promoting the terminal differentiation stage of osteoblast. SATB2 inhibits Hoxa2 activity in the early stage of osteoblast differentiation. Runx1 plays an important role in inhibiting adipocyte differentiation and promoting chondrocyte differentiation. The interaction between osteoblasts and osteoclasts is also very important. Osteoblasts regulate osteoclast differentiation via RANKL signaling and inhibit osteoclast differentiation through OPG. Similarly, osteoclasts can regulate osteoblast differentiation through the Wnt10b, BMP6, or Ephrin signaling pathway.

**Fig. 2:**
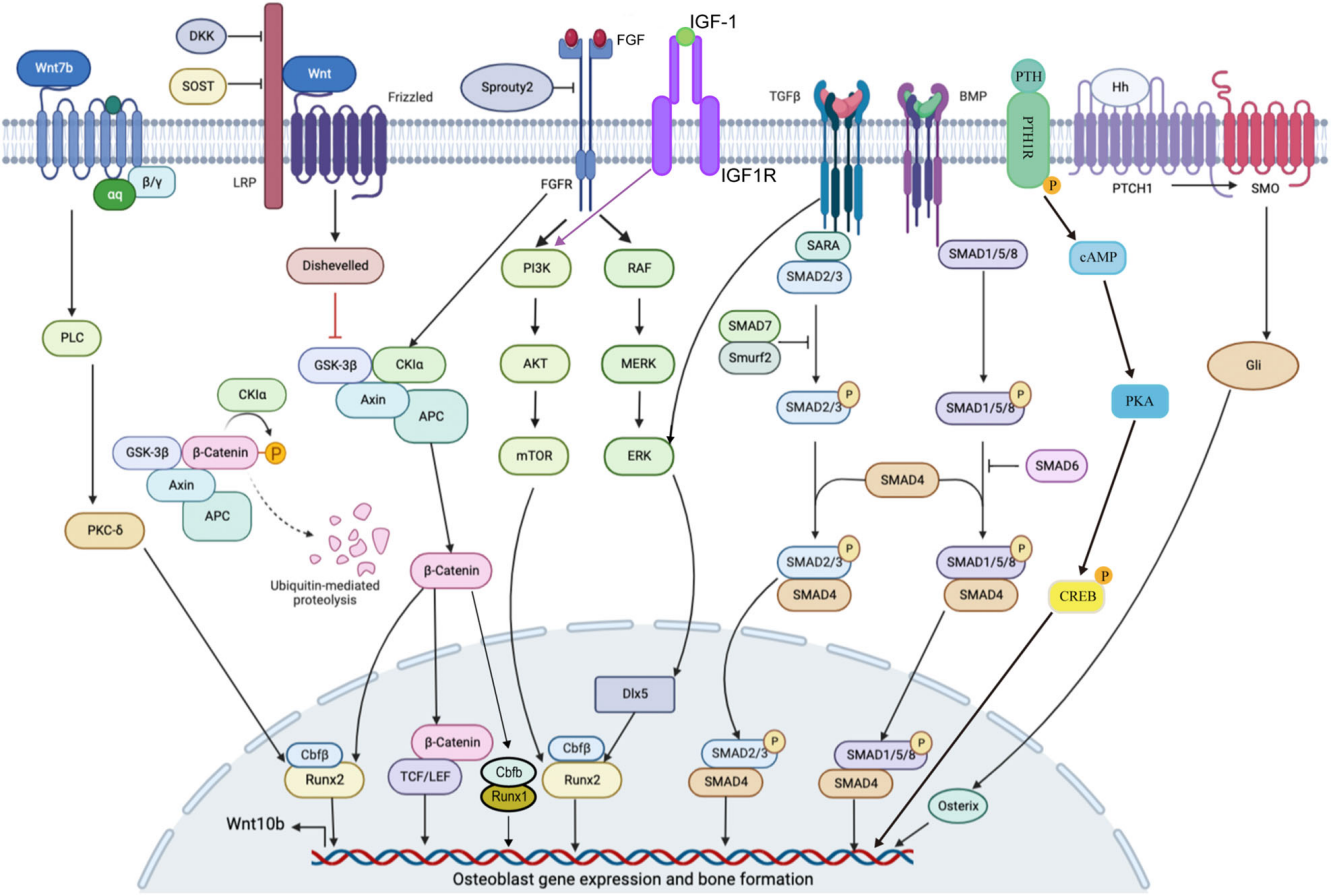
Canonical signaling pathways in osteoblast differentiation. Several canonical signaling pathways control the activity of key transcription factors to mediate osteoblast differentiation. Wnt, TGF-β, BMP, FGF, and Hedgehog pathways are the most classic pathways that have been studied during osteoblast differentiation. Wnt binds with FZD receptors, causing the β-catenin accumulation. β-catenin then moves to the nucleus, in which it causes target genes to be transcribed. Wnt signaling also regulates Runx1 and Runx2 functions. TGF-β and BMP signaling regulate osteoblast-specific gene expression through multiple Smad proteins. TGF-β signaling mainly activates Smad2/3, while BMP signaling activates Smad1/5/8. FGF and FGFR can also regulate osteoblast differentiation and osteoblast-specific gene expression via downstream pathways such as PI3K-AKT and ERK pathways. Runx2, Osterix, and several other transcription factors are also necessary for osteoblast differentiation, and these transcription factors are regulated by these classic pathways. Hedgehog signaling is activated through Hh ligand binding to the 12-transmembrane receptor Patched 1 (PTCH1), which relieves inhibition of the seven-pass transmembrane G protein-coupled receptor Smoothened (SMO). Actived SMO can initiate the intracellular cascade leading to the activation of three Gli transcription factors, and Gli can then translocate into the nucleus and regulate Osx activation, thereby modulating osteoblast-specific gene expression. PTH regulates osteoblast differentiation by binding with PTH1R, subsequently activating cAMP and PKA, leading to the phosphorylation of CREB to regulate osteoblast-specific gene expression. IGF-1 binds to its receptor IGF1R and activates PI3K-Akt pathway, resulting in the activation of mTOR and promotion of osteoblast differentiation.

Second, osteoclast differentiation can be directly regulated by osteoblast protein expression^3^. For example, macrophage colony-stimulating factor (M-CSF) and receptor activator of nuclear factor kappa β ligand (RANKL), two fundamental proteins for osteoclast differentiation, can be expressed by osteoblasts (Fig. 1)^4^. Osteoblast-produced M-CSF and RANKL have the ability to bind to C-FMS and RANK receptors (respectively) on osteoclast progenitors, triggering subsequent signals that promote osteoclast differentiation by activating the transcription factor nuclear factor of activated T cells 1 (NFATc1)^5^. Osteoblasts secrete osteoprotegerin (OPG) and can regulate osteoclasts by binding to RANKL, preventing RANKL from binding to RANK. Chemoattractants (CCL8, CCL6, and CCL12) that can influence osteoclast differentiation and recruit osteoclast precursors to bone are also regulated by osteoblast calcineurin/NFAT signaling^6^. Osteoclasts can also regulate osteoblast differentiation. Previous work in our had found that osteoclasts regulate osteoblast differentiation and tooth root formation via IGF/AKT/mTOR signaling^7^ (Fig. 2).

Third, osteoblasts are inextricably linked to the behavior and function of HSCs^8^. Research has demonstrated the significant involvement of osteoblasts in the HSC microenvironment^9–11^. Based on studies of PTH/parathyroid-related protein receptor (PPR) and bone morphogenetic protein receptor 1a (Bmpr1a), osteoblasts that express N-cadherin form N-cadherin/β-catenin adhesion complexes with HSCs^9^. Osteoblasts also produce many essential molecules for HSC renewal and maintenance, such as osteopontin (OPN), thrombopoietin, annexin-2, and angiopoietin-1^12,13^. Similarly, C-X-C Motif Chemokine Ligand 12 (CXCL12) in mesenchymal progenitor cells is required for the maintenance of HSCs and can maintain HSCs in an undifferentiated state^14^. These complexes may mediate the adhesion of hematopoietic stem cells within their niche^15^.

## Osteoblast origin and cell lineage

The process of osteoblast formation involves a series of distinct stages and cell types within the osteoblast lineage. Osteoblast lineage cells include mesenchymal progenitors, pre-osteoblasts, osteoblasts, osteocytes, and bone-lining cells (Fig. 1). The exact origin of osteoblasts can vary depending on the life stage and the specificity of the local environment, and therefore, the naming of progenitor cells has always been controversial^16^. Skeletal stem/progenitor cells (SSPCs) can broadly include all immature precursor cells located within the bone and can differentiate into bone tissue-forming cell types^16^. This includes cartilage stem cells, recently found in growth plates^17,18^, fetal perichondrium cells which give rise to bone lines^19^, and periosteum progenitor cells, in endochondral bone development which is important in fracture repair^20,21^, as well as distinct reticular and perivascular stromal cell subpopulations in bone marrow, commonly referred to as bone marrow stromal cell (BMSC) populations, which seem to have lipogenic potential in some cases^22,23^.

During development, osteoblast formation proceeds via two distinct pathways: intramembranous ossification or endochondral ossification (Fig. 1). In mammals, intramembranous ossification primarily affects only a portion of the clavicle and the skull, whereas endochondral ossification controls bone formation throughout the skeletal system^24^. During intramembranous ossification, MSCs can directly differentiate into osteoblasts, while osteoblasts are indirectly formed during endochondral ossification through the formation of the periosteum, which contains immature osteoprogenitor cells^25^. Osteoblastogenesis during intramembranous ossification occurs through three main stages: mineralization, proliferation, and matrix maturation. On the other hand, endochondral ossification occurs in two steps: building cartilage templates in a nonvascularized environment and steadily replacing the cartilage template with bone. Through mesenchymal condensations, endochondral ossification constructs a framework for subsequent bones and leads to the mass production of undifferentiated mesenchymal cells. Following the action of SOX9, condensing mesenchymal cells quickly transform into chondrocytes (Fig. 1), which will continue to proliferate and produce cartilage templates^26^. At this stage, the perichondrium forms a highly vascularized fibrous tissue adjacent to the cartilage template. In an area immediately adjacent to the pre-hypertrophic zone, the perichondrium produces the first osteoblasts in endochondral bones. Later, the perichondrium will develop into the bone collar and periosteum^27^.

During intramembranous ossification, lineage commitment of MSCs is dependent on the early activation of specific BMPs, peroxisome proliferator-activated receptor γ (PPAR-γ), and Wnt signaling, with alkaline phosphatase (ALP), collagen type I (COL1A1), OPN, osteocalcin (OCN), and PPR among the markers most highly expressed during the differentiation of osteoblasts (Table 1; Fig. 1)^15,25,28^. After initial lineage commitment, an MSC will divide into an osteoprogenitor and an additional stem cell; this division is important to maintain frequent proliferative activity and self-renewal^29^. The newly formed pre-osteoblast is an intermediate cell that can express the MSC marker STRO1, ALP, PPR, and collagen type I and possesses an extensive capacity for replication; however, it has no capacity for self-renewal^30^. Located near newly synthesized osteoid cells, ALP, OPN, bone sialoprotein (BSP), and OCN are expressed by mature osteoblasts. The pre-osteoblast cell is responsible for bone deposition and has limited replication potential^31^. Terminal differentiation and permanent stoppage in cell division are the next crucial steps. Post-mitotic osteocytes are the final stage of the bone lineage. They are typically found separated in the bone and may be embedded in developing osteoid cells.

## Osteoblast signaling

**Fig. 3:**
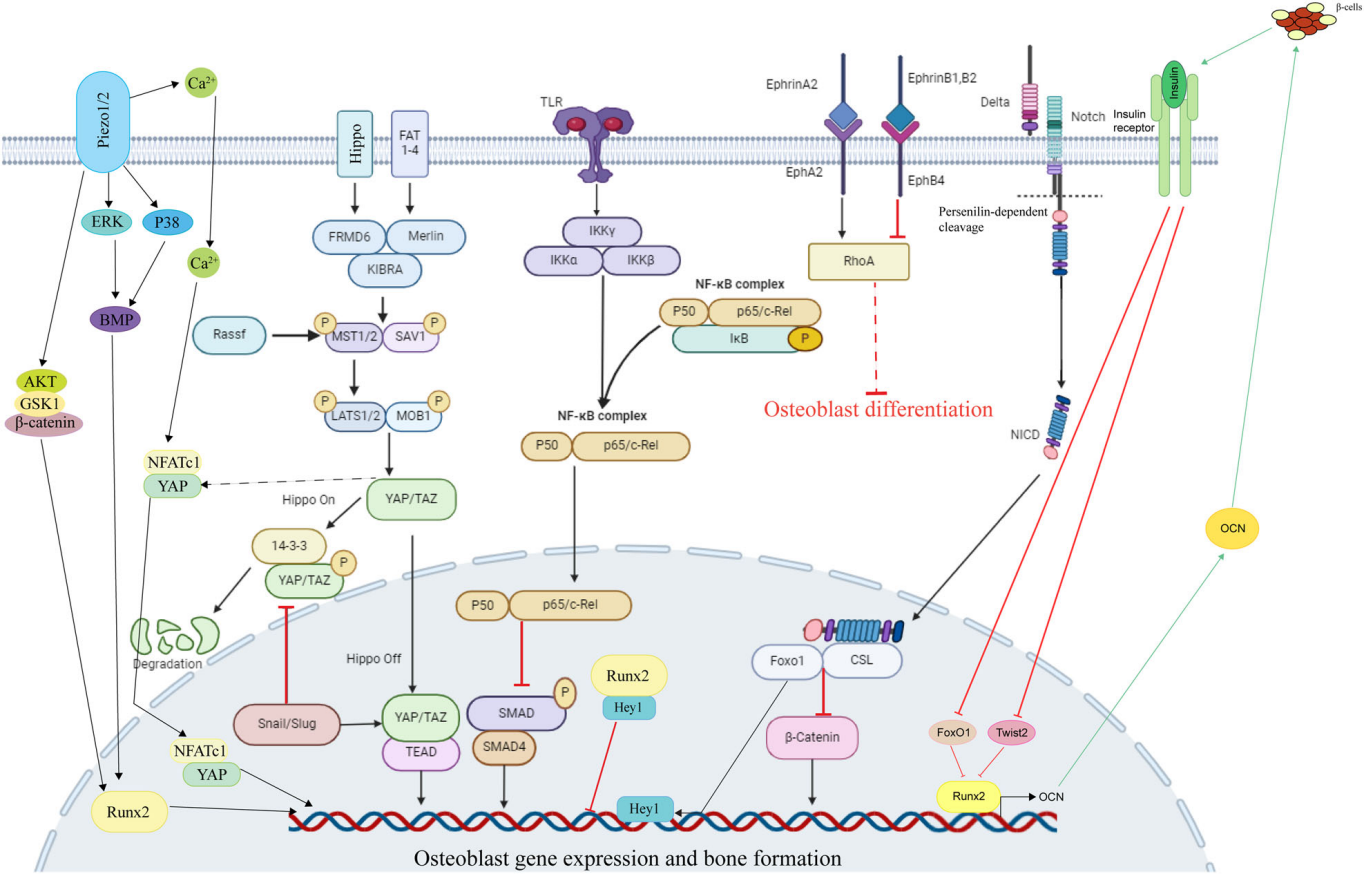
Noncanonical signaling pathways in osteoblast differentiation. Notch, NF-κB, Hippo, Ephrin, and Piezo1/2 signaling pathways also play important roles in osteoblast differentiation. Osteoblast differentiation is inhibited by the Notch and NF-κB signaling pathways, with the Notch signaling pathway’s downstream factors NICD binding to CLS and inhibiting β-catenin. NICD/CLS/Foxo1 complex can promote Hey1 expression, then Hey1 can bind with Runx2 to inhibit Runx2 activity. When NF-κB signaling is stimulated, the P50 and P65 complex translocates into the nucleus and inhibits Smad protein activity. Hippo signaling is a crucial pathway in cell growth and development. The important downstream factors YAP/TAZ in Hippo signaling can regulate osteoblast differentiation-related gene expression. When Hippo signaling is in an “off” state, the YAP/TAZ complex will not be degraded and will translocate into the nucleus to regulate osteoblast-specific gene expression. The snail/slug complex can promote YAP/TAZ activity in nuclear and inhibit YAP/TAZ degradation in the cytoplasm. Ephrin signaling has different regulatory effects on osteoblast differentiation through different pathways and plays a specific role in the regulation of osteoblasts and osteoclasts. The EphrinA2 and EphrinB2 expressed on osteoclast membranes can interact with EphA2 and EphB4 expressed on osteoblast membranes to regulate osteoblast differentiation. Ephrin signaling regulated osteoblast-specific gene expression mainly through RhoA. EphrinA2 binds with EphA2 and promotes RhoA activity, while EphrinB1/2 binds with EphB4 and inhibits RhoA activity. When RhoA is activated, it will inhibit osteoblast differentiation. Piezo1/2 is a recently discovered pathway functioning in osteoblast differentiation. Piezo1/2 can regulate osteoblast-specific gene expression by regulating downstream pathways such as ERK and P38 and interacting with Hippo signaling. Piezo1/2 can regulate β-catenin activation to active Runx2 and regulate osteoblast gene expression. Piezo1/2 can also activate NFATc1 and YAP via calcium signal, and then NFATc1 and YAP can translocate into the nucleus and regulate osteoblast differentiation. Insulin signaling inhibits the production of FoxO1 and Twist2, which can inhibit the expression of Runx2 and Ocn. Ocn improves insulin sensitivity and energy expenditure through multiple mechanisms like activating to β-cells. The direct effect of osteocalcin as an insulin-sensitizing hormone is speculative and remains to be determined.

Several essential signaling pathways regulate osteoblast differentiation (Figs. 2, 3). Wnt, TGF-β, Hedgehog, and FGF signaling pathways are canonical pathways known to affect osteoblast differentiation. Additionally, Notch, Hippo, and NF-κB signaling also affect osteoblast differentiation.

### Wnt signaling pathway

#### Overview of Wnt signaling

The family of secretory glycoproteins known as Wnt contains numerous protein factors that are ligands of the 7-membrane-spanning frizzled (FZD) receptor family (Fig. 2). The secreted factors of the Wnt family are implicated in numerous cellular processes, such as regulating cell polarity, cell differentiation, cell proliferation and cell function. There are two different categories of Wnt proteins. The first type causes activation of canonical Wnt signaling by forming complexes among Wnt proteins, the low-density lipoprotein receptor-associated protein 5 or 6 (LRP5 or LRP6) and FZD^32,33^ (Fig. 2). Of note, LRP4 has been implicated in sclerosteosis and van Buchem disease due in part to its role in Wnt signaling, with deficiency of LRP4 in osteoblast-lineage cells causing higher cortical and trabecular bone mass in mutant mice, which is linked to increased bone formation and less bone resorption^34^. The second type of Wnt protein causes activation of the noncanonical pathway through a protein kinase C-dependent mechanism, where Wnt5a binds with FZD to activate heterotrimeric G proteins and subsequently raises intracellular calcium to promote osteoblast function^35^.

**Fig. 4:**
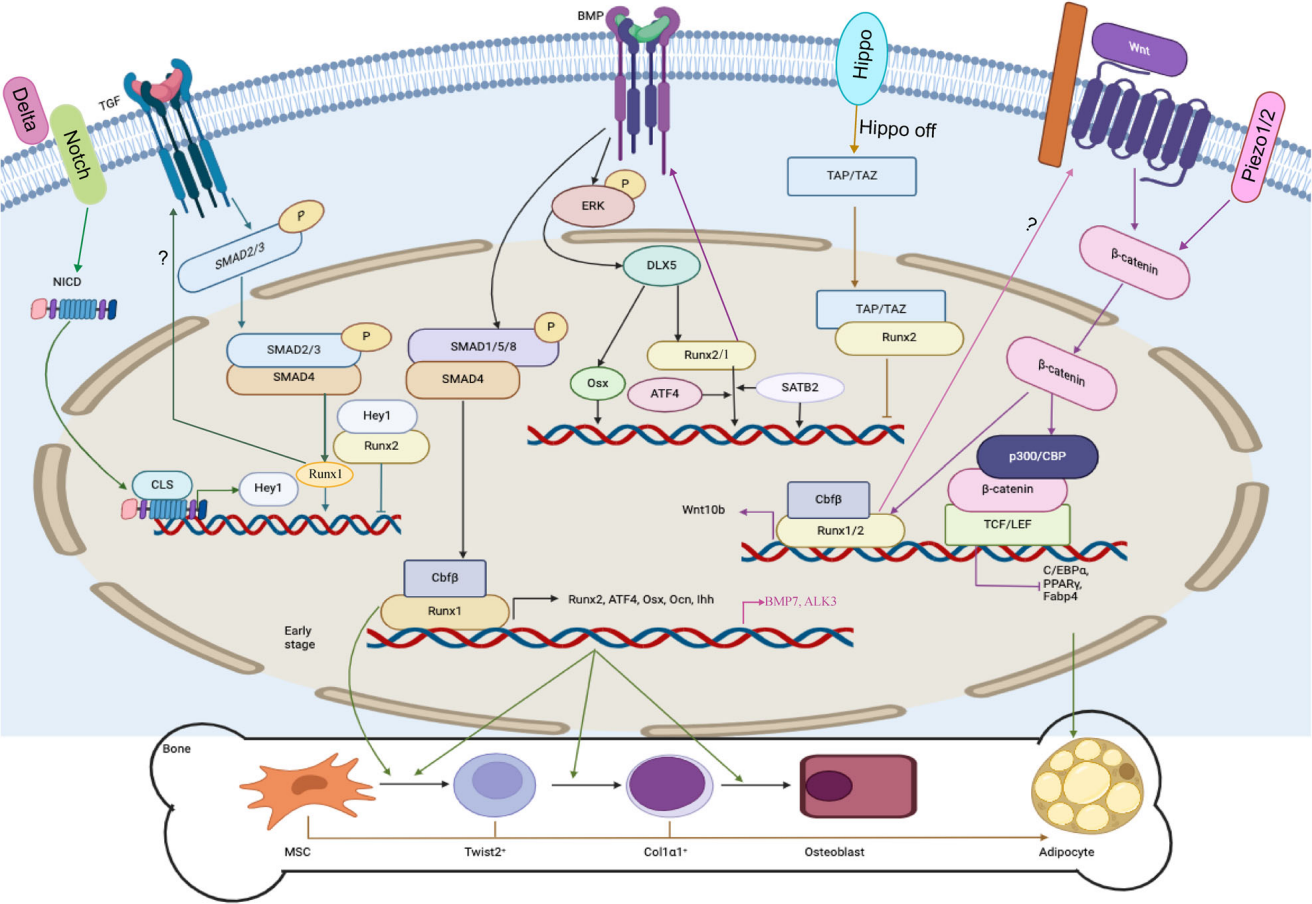
Signaling and transcriptional regulation of osteoblast cell lineage commitment, differentiation, and bone formation. Among the many transcription factors that participate in osteoblast differentiation, the Runx family, Osx, ATF4, Cbfβ, and SATB2 are significant. Cbfβ binds to Runx family proteins to form heterodimers, improving Runx2’s and Runx1’s stability and subsequently facilitating Runx2 or Runx1 binding to target DNA sequences. Runx1 positively regulates osteoblast lineage gene expression at various stages of differentiation. Runx1 plays a significant role in postnatal bone homeostasis by binding to ATF4, Ocn, and Runx2 promoters to activate the corresponding genes and promote osteoblast early differentiation. Runx1 promotes BMP7 and Alk3 expression to regulate BMP signaling. How Runx1 can regulate Wnt10b and TGF-β signaling remains unclear. Runx1 can also regulate osteoblast-adipocyte lineage via inducing Wnt/β-catenin signaling, TGF-β signaling, and restraining adipogenic gene transcription. Cbfβ is also crucial in stimulating osteogenesis by inhibiting the expression of the adipogenesis regulatory gene C/EBPα and activating Wnt10b/β-catenin signaling. Wnt binds with FZD receptors, causing the ß-catenin accumulation. Pizeo1/2 can also regulate ß-catenin. ß-catenin then moves to the nucleus, in which it causes target genes to be transcribed by interacting with P300/CBP and TCF/LEF. TGF-β and BMP regulate transcription factors through SMAD proteins to activate Runx1 and Runx2 activity, and the SMAD itself can also regulate osteoblast-specific gene expression. BMP signaling can also activate Dlx5 through ERK and promote Runx2, ATF4, and Osx expression. When the Hippo signaling is at the “off” state, YAP/TAZ can translocate into the nucleus and bind with Runx2 to inhibit its activity. NICD1 in Notch signaling can translocate into the nucleus and promote the expression Hey1, which in turn inhibits Runx2 function by binding with Runx2. The activation of β-catenin and Runx2 will also inhibit the expression of C/EBPα, PPAR-γ, and Fabp4; these genes are important in adipocyte differentiation.

Both canonical and noncanonical Wnt signaling are crucial for bone remodeling^36,37^. Wnt proteins, whether displayed on the cell surface or secreted, have the capability to engage osteoblasts via binding with the FZD/LRP5/6 complex^38^. The PTH1 receptor, which binds PTH and forms a complex with LRP5/6, can also activate the Wnt signaling in the absence of the Wnt ligand. Disheveled, Axin^39^, and Frat-1 proteins stimulate the signal by destroying the protein complex and inhibiting glycogen synthase kinase 3 (GSK3) function, resulting in reduced phosphorylation of β-catenin^40^ (Fig. 2). By hindering GSK3 activity, the stability of β-catenin is improved and Wnt signaling is turned on (Fig. 2). MiR-346 promotes osteoblast differentiation by inhibiting glycogen synthetase kinase 3β (GSK-3β), preventing β-catenin degradation in human BMSC^41^ (Fig. 2). Recent studies have shown that 1-Azakenpaullone, a highly selective GSK-3β inhibitor, is a potent inducer for osteogenic differentiation and mineralization of human MSCs^42^. 1-Azakenpaulone significantly induces osteogenic differentiation and mineralization of human MSCs by activating Wnt signaling, leading to the up-regulation of Runt-related transcription factor 2 (Runx2), a key transcription factor that ultimately promotes osteogenic specific gene expression^42^. Once stabilized in the cytoplasm, β-catenin translocates to the nucleus to promote target gene expression. The nuclear partners of β-catenin are the lymphoid enhancer-binding factor/T cell factor (Lef/Tcf) family^43,44^. β-catenin substitutes Lef/Tcf corepressors, establishing heterodimers with Lef/Tcf proteins (Fig. 2). With the help of P300/CBP and other transcriptional co-activators, this heterodimer binds to DNA and promotes target gene transcription for osteoblast differentiation^45^ (Fig. 4).

β-catenin degradation can be facilitated through its interaction with protein complexes consisting of APC, Axin, and GSK3 if Wnts are either not expressed or are unable to bind to the receptor (Fig. 2). GSK3 phosphorylates β-catenin, after which the phosphorylated form undergoes ubiquitination by the β-TrCP ubiquitin E3 ligase and subsequent degradation via the ubiquitin-dependent proteasomal system^46^ (Fig. 2). Pannexins3 (Panx3) can promote the degradation of β-catenin by activating GSK3β, thus inhibiting the activity of the Wnt/β-catenin pathway and the differentiation process of osteoblasts^47^. In the nucleus, Tcf/Lef and its repressor bind to inhibit target genes downstream of the Wnt signaling. In the “off” state of Wnt signaling, characterized by low cytosolic and nuclear levels of β-catenin, osteoblast differentiation is reduced^44^ (Fig. 2). Similarly, promotion of APC degradation via genistein-induced autophagy initiated Wnt/β-catenin signaling pathway activation and β-catenin–driven osteoblast differentiation, rebalancing dysregulated osteogenesis within the femurs and tibias of OVX mice^48^. Taken together, these findings could indicate that targeting the degradation mechanisms for β-catenin may be a potential avenue for better treatment of diseases driven by improper osteoblast differentiation.

Wnt ligands can bind with a variety of inhibitors to limit pathway activation, like FZD-associated protein (sFRP) secretion and Wnt repressor SOST (Sclerosteosis gene product)^49^, Dickkopf (Dkk) family^50^, and Src (Fig. 2). These inhibitors like SOST and Dkk can bind to LRP5/6 and inhibit its coreceptor activity. Capable of interfering with canonical Wnt signaling, Dkk1 and Dkk2 bind to LRP5/6 and kremen (a transmembrane protein) (Fig. 2)^51^. The silencing of the FZD6 receptor influences osteoblast differentiation, restraining osteoblast differentiation through the canonical pathway of Wnt. Meanwhile, FZD4 silencing reduces the rate of osteoblast differentiation via reduced expression of Tcf/Lef^52,53^.

Conversely, the promotion of osteoblast differentiation through the stabilization of Wnt-signaling can be achieved through various mechanisms. The R-spondin family is comprised of four secreted glycoproteins (Rspo1–4) and functions to amplify Wnt signaling. Notably, Rspo3 variants are strongly linked to bone density^54,55^. A recent study indicated that in appendicular bones, insufficient Rspo3 haploid and loss of Rspo3 targeting in Runx2^+^ bone progenitor cells can lead to increased trabecular bone mass, osteoblasts, and bone formation numbers^54^. The inhibitory effect of Dkk1 on Wnt signaling activation and bone mass is compromised by Rspo3 deficiency^54^. Defects in Rspo3 result in the activation of extracellular signal-regulated kinase (ERK) signaling, which subsequently stabilizes β-catenin and activates Wnt signaling, thereby promoting osteoblastogenesis and bone formation^54^.

Wnt signaling performs various functions in different stages of osteoblast differentiation. Canonical Wnt signaling can enhance early osteoblast differentiation, while mature osteoblast mineralization induction is severely hampered by it^56,57^. During osteoblast differentiation, multiple Wnt signaling genes are changed, and endogenous Wnt signaling is suppressed^58,59^. Wnt16 can regulate Wnt/β-catenin signaling by antagonizing Wnt5a to suppress the non-canonical Wnt/Ca^2+^ pathway^60^. In a prior study, it was discovered that Wnt16 enhances the expression of osteoblast differentiation genes (BMPR1b, BMP7, and ENPP1), while simultaneously reducing the expression of specific osteoblast maturation and mineralization genes (ALP1 and RSPO2)^61^. However, another study found that Wnt16 positively regulates osteoblast differentiation and matrix mineralization^60^. Therefore, the mechanistic basis for how Wnt16 regulates osteoblast mineralization still needs further research.

#### Wnt canonical pathway

The canonical Wnt/β-catenin signaling pathway promotes bone formation by stimulating osteoblast development^62–64^. Bone formation and specific osteoblast gene expression are both enhanced by high levels of β-catenin^62,65^, while ectopic chondrogenesis and abnormal osteoblast differentiation resulted from conditional β-catenin knockdown at the initial stage of osteoblast development^65–67^ (Table 1). Additionally, Wnt is involved in all stages of bone development, as demonstrated by the process of bone formation exhibited by LRP5-deficient mice^68–70^. Altered bone mass was observed in mice with LRP5 deficiency^71,72^, indicating that Wnt signaling is crucial during bone development^73,74^. Furthermore, the functional acquisition of the LRP5 mutation increases Wnt signaling, leading to higher bone mineral density in mammalian models^73,75–77^ (Fig. 2).

Wnt-LRP5 signaling promotes bone formation in part by directly reprogramming glucose metabolism^78^. Mammalian target of rapamycin complex 2 (mTORC2) and protein kinase B (AKT) are activated by Wnt3a via LRP5 and ras-related C3 botulinum toxin substrate1 (RAC1), which causes the activity of glycolytic enzymes to increase^78^. In addition, the Warburg effect, induced by Wnt3a’s elevation of key glycolytic enzyme levels, can trigger aerobic glycolysis^78^. In vitro metabolic regulation aids Wnt-induced osteoblast differentiation and is linked to the bone-forming activity of LRP5 signaling in vivo^78^. Exosomes of adipose stem cells (ASCs-exos) have recently been found to promote osteogenic differentiation of BMSCs, with ASCs-exos improving bone healing ability in a rat model of nonunion fracture repair^79^. ASCs-exos play a role in activating the Wnt3a/β-catenin signaling pathway and promoting osteogenic differentiation of BMSCs^79^. By activating the Wnt3a/β-catenin signaling pathway, ASCs-exos enhance the osteogenesis potential of BMSCs and promote bone repair and regeneration in vivo, providing a new direction for the treatment of diabetic fracture nonunion^79^.

Bipotential mesenchymal precursors undergo osteoblastogenesis stimulation and adipogenesis inhibition via canonical Wnt signaling. By enhancing osteoclastogenic transcription factors like Runx2 and Osx while inhibiting adipogenic transcription factors, Wnt10b induces osteoblast differentiation^62^. The inhibition of PPAR-γ and C/EBPα expression is one way through which Wnt10b promotes osteoblastogenesis^62^. C/EBPα is also an essential regulator in osteoclast differentiation^80–82^. Indeed, Wnt10b^–/–^ mice showed reduced trabecular bone density and serum osteocalcin, further illustrating that Wnt10b is an endogenous regulator of bone formation^62^.

#### Wnt noncanonical pathway

Previous research has extensively studied how the canonical Wnt signaling pathway can influence osteoblast differentiation. However, the noncanonical Wnt signaling pathway also plays a significant role in mediating osteoblast differentiation. In comparison to canonical Wnt signaling, the non-canonical Wnt signaling pathway regulates osteoblast differentiation not through β-catenin but rather through various other transcriptional and signaling mechanisms.

##### Wnt5a

Wnt5a is a significant marker of noncanonical Wnt signaling, with Wnt5a inhibiting the expression of PPAR-γ through SET domain bifurcated histone lysine methyltransferase 1 (SETDB1) to promote osteoblast differentiation^83^. Although TAZ (Table 1) is an important transcription factor that promotes MSC differentiation by trans-inhibiting PPAR-γ function, noncanonical Wnt signaling via Wnt5a activation does not appear to modulate TAZ expression during osteoblast differentiation^83^. Following Wnt5a-induced activation of noncanonical Wnt signaling, SETDB1 interacts with chromodomain-helicase-DNA-binding protein 7 (CHD7) and phosphorylated nemo-like kinase (NLK) to form a multi-protein complex^83^. This complex methylates the histone H3 lysine 9 (H3K9) of the PPAR-γ promoter, resulting in chromatin inactivation and the failure of PPAR-γ to initiate transcription^83^. By suppressing the transformation of bone marrow MSCs into adipocytes via a reduction in PPAR-γ’s transcriptional effect, Wnt5a can stimulate osteoblast production^83^.

##### Wnt/calcium pathway

In the process of osteoblast differentiation, the Wnt/calcium pathway operates as a noncanonical Wnt signaling pathway. The Wnt/calcium pathway can raise intracellular calcium levels to activate calcineurin, protein kinase C (PKC), and calmodulin-dependent protein kinase II (CaMKII), which in turn induces the expression of activating protein 1 (AP-1) transcription factors. The combination of integrin receptors and collagen I may cause CaMKII to be activated in osteoblasts, resulting in ERK phosphorylation, thus promoting Runx2 activation and subsequent osteoblast differentiation^84^. Therefore, the noncanonical Wnt signaling pathway can also promote osteoblast differentiation by regulating the expression of Runx2, which is mechanistically similar to canonical Wnt/β-catenin signaling.

##### Wnt7b

In osteoblast precursors, the noncanonical Wnt pathway plays a role in promoting bone formation. This involves Wnt-mediated activation of G protein-linked phosphatidylinositol signaling, leading to the activation of G protein-linked protein kinase C δ (PKCδ). This process, facilitated by Dvl, remains unaffected by Dkk1^85^. In mouse embryos, the elimination of either Wnt7b or PKCδ led to impaired bone formation^85^. Wnt 7b induces osteoblast differentiation via PKCδ (Fig. 2)^85^. Wnt-induced PKC activation requires the Gq subunit, which is required for osteoblast differentiation induced by Wnt7b. Wnt7b activates Runx2 through PKCδ signaling to regulate osteoblast-specific gene expression. Although PKCδ signaling activates osteoblast differentiation, in vitro experiments demonstrated that Wnt7b or PKCδ mutant mice can still form bone^85^. This suggests that this pathway can act as a mechanism to augment other osteogenic signaling pathways, but it may not be enough for osteoblast differentiation^85^.

Prior research has suggested a connection between increased glycolysis and osteoblast differentiation triggered by Wnt signaling^86^. However, direct genetic confirmation of the role of glucose metabolism in Wnt-induced bone formation has been lacking. A recent study revealed that overexpression of Wnt7b significantly enhanced bone formation, yet this effect was largely negated in the absence of Glucose Transporter 1 (Glut1), despite the transient deletion of Glut1 itself not affecting normal bone accrual^87^. Wnt7b was also observed to elevate Glut1 expression and glucose consumption in primary cultures of osteoblast lineage cells, with Glut1 deletion impairing osteoblast differentiation in vitro^87^. Thus, Wnt7b appears to contribute to bone formation partly by stimulating glucose metabolism in osteoblast lineage cells^87^.

#### Role of β-catenin in osteoblast differentiation

β-catenin is required for mature osteoblast differentiation (Table 1)^65,66,88^. Without β-catenin, osteoblast progenitors will instead develop into chondrocytes, with chondrogenic interstitial precursor fate being determined by β-catenin activity^65,66^. Runx2 promoter activity and expression are both enhanced by β-catenin/TCF1^89^. Experimental findings have shown that mice lacking β-catenin develop osteopenia (Table 1), while bone mass was increased when β-catenin function was rescued in osteoblasts^67,90^. Previous work in our lab also found that Cbfβ/Runx2 complex promotes Wnt10b/β-catenin to improve osteoblast differentiation and inhibit c/ebpα expression to inhibit lineage switch into adipocytes^91^.

#### Regulation of Wnt signaling pathway

MTSS1 plays a significant role in osteoblast differentiation and bone homeostasis by controlling Src-Wnt/β-catenin signaling. In osteoblast differentiation, Src acts as a negative regulator by phosphorylating LRP6 at several conserved tyrosine residues. This phosphorylation disrupts the removal of LRP6 from the osteoblast cell surface and the formation of LRP6 signalosomes, thereby inhibiting canonical Wnt signal transduction^92^. When MTSS1 increases, Src diminishes the content of phosphorylated-GSK3β, non-phospho-β-catenin, and transcription factor 7 like 2 (TCF7L2)^93^. Thus, Src inhibits Wnt/β-catenin signaling activation in osteoblasts, whereas MTSS1 stimulates Wnt/β-catenin signaling by inhibiting the non-receptor tyrosine kinase Src.

Similar to MTSS1, Tenascin-C also positively regulates Wnt signaling. Deletion of Tenascin-C significantly inhibits osteoblast differentiation, indicating its role as a positive regulator of Wnt signaling^94^. Likewise, β-catenin overexpression can significantly reverse the inhibition of osteoblast differentiation that is caused by Tenascin-C deficiency^94^. Tenascin-C can bind to DKK-1, the primary inhibitor of Wnt signaling, suppressing DKK-1 function^94^. Previous work has shown that DKK-1 expression is increased when Tenascin-C is knocked down by siRNA, thereby reducing the transcriptional activity of Wnt signaling^94^.

TNF receptor-associated factor 3 (TRAF3), a TNF receptor family adaptor protein, positively regulates osteoblast differentiation^95^. Mice deficient in TRAF3 exhibited reduced bone formation and increased bone resorption, displaying a phenotype indicative of early-onset osteoporosis^95^. It was also discovered that TRAF3 promoted osteoblast formation and prevented the degradation of β-catenin in MSCs^96^. TGF-β1, which is released when bone resorption occurs, induces TRAF3 degradation and thus inhibits osteoblast differentiation through GSK-3β-mediated β-catenin degradation^96^.

Hormones can regulate the canonical Wnt signaling pathway by controlling important proteins in Wnt signaling. In osteoblast progenitors that express Osterix1 (Osx1), Wnt/β-catenin signaling is boosted by estrogen receptor α (Erα), causing an increase in periosteal cell proliferation and differentiation^97^. Estrogen-related receptor α (ERRα), in conjunction with the coactivator peroxisome proliferator-activated receptor-gamma coactivator (PGC-1α), acts as a positive regulator of Wnt signaling in osteoblast differentiation. This occurs via a cell-intrinsic mechanism that remains unaffected by β-catenin nuclear translocation^98^. Activated ERRα can bind with the TCF/LEF complex and stimulate osteoblast-specific gene expression similar to that induced by β-catenin^98^. Thus, it can be understood that TCF/LEF is activated to promote the expression of osteoblast-specific genes in the absence of β-catenin nuclear translocation.

### TGF-β and BMP signaling

TGF-β and bone morphogenetic proteins (BMPs) are members of the TGF-β superfamily, playing crucial roles in osteoblast differentiation^99,100^. There are two main types of TGF-β and BMP signaling: one is Smad-dependent and the other is Smad-independent (Fig. 2)^101^. There are three Smad types. The first is the receptor-regulated Smads (R-Smads), which can either be triggered by BMPs (Smad1/5/8) or activated by TGF-β (Smad2/3). The second is co-Smads, such as Smad4, which can be co-mediated by BMP and TGF-β. The third is inhibitory Smads, like Smad6 and Smad7, which can negatively regulate BMP and TGF-β signaling (Fig. 2)^102,103^. R-Smads and co-Smads combine to form a complex that moves to the nucleus and controls osteoblast-specific gene expression^101^. Runx2 expression is not directly induced by the BMP signaling pathway^104^, but it can be induced by promoting distal-less homeobox 5 (DLX5) expression (Fig. 2)^105,106^.

#### Overview of TGF-β signaling

TGF-β signaling pathways govern osteoblast and chondrocyte differentiation, influencing bone formation at different stages of development and within disease pathology. Smad proteins are critical in TGF-β signaling pathways (Fig. 2)^102,107^. In conjunction with Smads, TGF-β signaling regulates osteoblast and chondrocyte differentiation^108,109^. TGF-β signaling primarily activates Smad2/3 to regulate osteoblast differentiation. However, TGF-β can also bind to ALK-1, transducing SMAD-1, 5, and 8 signaling, typically activated by BMPs (Fig. 2)^110,111^. During the early stage of differentiation, TGF-β signaling promotes osteoprogenitor proliferation and osteogenesis. However, at later stages, it inhibits bone formation^102^. Previous work in our lab also found that TGF-β1 signaling pathway also is critical in tooth root development and odontoblast differentiation^112^. Moreover, in vitro studies have demonstrated that TGF-β, along with SMAD3 and SMAD2, inhibits osteogenesis^113–116^. Excessive TGF-β signaling was also found to be involved in the pathogenesis of osteogenesis imperfecta in mouse models, with anti-TGF-β treatment correcting altered bone phenotype^117^. These findings were validated in a small clinical study, where the administration of the monoclonal antibody fresolimumab to participants showed an absence of severe side effects. Furthermore, it was noted to enhance the areal bone mineral density in the lumbar spine of individuals diagnosed with osteogenesis imperfecta type IV^118^. Deletion of Transforming growth factor beta receptor 2 (TGFBR2), the sole type II receptor for TGF-βs, effectively abolishes TGF-β signaling. This deletion leads to severe defects in calvarial, appendicular, and axis bones^119^.

#### TGF-β signaling regulation

Higenamine (HG) has been recognized as a promising candidate for treating osteoporosis through its influence on SMAD2/3 signaling. A novel target for HG has been identified in IQ motif-containing GTPase activating protein 1 (IQGAP1), with HG binding to the Glu-1019 site of IQGAP1, facilitating its osteogenic effects^120^. Through this mechanism, HG induces Smad2/3 phosphorylation and modulates the Smad2/3 pathway by inhibiting Smad4 ubiquitination^120^. Consequently, HG emerges as a potential novel small-molecule drug to stimulate bone formation in osteoporosis via the Smad2/3 pathway^120^.

Recent research has suggested that intraflagellar transport 20 (IFT20) modulates osteoblast differentiation and bone formation by regulating the TGF-β signaling pathway^121,122^. IFT20 controls MSC lineage allocation by regulating glucose metabolism during bone development^123^. In MSCs, the absence of IFT20 results in a significant reduction in glucose tolerance and inhibits glucose uptake, lactic acid production, and ATP production^123^. Deleting IFT20 markedly reduced the signaling activity of TGF-β-Smad2/3, decreased the binding activity of Smad2/3 to the Glut1 promoter, and down-regulated Glut1 expression^123^. These findings suggest that IFT20 plays a vital role in preventing the allocation of MSC lineages to fat cells through the TGF-β-Smad2/3-Glut1 axis^123^. Moreover, GATA binding protein 2 (GATA2), necessary for HSC differentiation, can impede osteoblast differentiation by inhibiting Smad1/5/8 activation^124^.

### Overview of BMP signaling

BMP signaling (an evolutionarily conserved group of signaling proteins belonging to the TGF-β superfamily) also has an effective role in osteoblast differentiation. Indeed, BMPs display effective osteogenic effects, similar to the physiological effect mediated by TGF-β^125^. BMP-2, -4, and -6 are signaling molecules that are highly expressed in both osteoblast cultures and bone tissue^126^. BMP molecules like BMP-2, -6, -7, and -9 aid in bone formation^127^. Of note, BMP3B negatively regulates bone formation^128^, in which BMP3B and BMP-2 may antagonize each other through competition with the availability of Smad4^129^.

Although BMP signaling can easily regulate the differentiation of osteoblasts through Smad proteins, it is also capable of this regulatory function by activating other signaling pathways controlled by Smad proteins. Prior research indicates that BMP-2 facilitates the interaction between Dvl-1 and Smad1, resulting in the inhibition of β-catenin nuclear accumulation^130^. Because Dvl-1 is an important inhibitor of GSK-3β, β-catenin is degraded when Dvl-1 binds to Smad1, thus inhibiting the activity of the Wnt signaling pathway^130^. BMP-2 can inhibit the proliferation of the Wnt signaling in this way, thus enabling MSCs to differentiate into osteoblasts^130^. Persicae semen (PS) originates from the dried and mature seeds of peach (*Prunus persica, L*.). PS facilitates mineralization and up-regulates Runx2 via BMP-2 and Wnt signaling pathways. Consequently, this leads to the expression of various osteoblast genes, including *Alp*, bone gamma-carboxyglutamate protein (*Bglap*), and integrin binding sialoprotein (*Ibsp*)^131^. Research has demonstrated that PS promotes fracture recovery by enhancing osteoblast differentiation and bone formation. Consequently, it could serve as a potential treatment option for patients with fractures^131^.

Aside from Smad-dependent pathways, BMP can regulate osteoblast differentiation through various other signaling mechanisms. BMP2 stimulates the expression of ALP and OCN by activating the mitogen-activated protein kinase (MAPK) signaling pathway^132,133^. By triggering Runx2 expression and activating c-Jun N-terminal kinase (JNK), BMP2 promotes osteoblast differentiation through the protein kinase D (PKD) pathway^134^. DLX5 has been shown to be a BMP2 signaling upstream target and can increase Runx2 expression downstream (Fig. 2)^135^. By activating DLX5 through the p38 signaling pathway, BMP2 can also induce Osx expression in addition to Runx2 expression (Fig. 2)^136^. In the bone microenvironment, BMP and other growth factors, like IGF-I, can co-activate Osx expression^137^. Recent research has also revealed that BMP2 can inhibit the expression and activity of salt-inducible kinase 1 (SIK1) through protein kinase A (PKA)-dependent pathways, thereby promoting osteogenesis^138^. SIK1 functions as a critical regulator, suppressing preosteoblast proliferation and osteoblast differentiation. The repression of SIK1 is crucial for facilitating BMP2 signaling in osteogenesis^138^.

#### BMP signaling regulation

The regulation of BMP signaling concerning osteoblast differentiation is influenced by numerous factors, all of which play distinctive roles in osteogenic processes. For instance, LIM homeobox transcription factor 1 beta (Lmx1b) hinders osteoblast differentiation by negatively controlling BMP2^139^. Lmx1b can decrease Runx2’s recruitment in the promoter sequence of the target gene and when combined with Runx2, inhibiting Runx2 activity^139^. Through the BMP2 promoter’s Tcf/Lef response elements, cWnt signals can trigger BMP2 activity^140^. BMP3 is a negative feedback regulator of osteoblast generation that is induced by cWnt, with decreased BMP3 expression possibly increasing the activity of the cWnt signal, encouraging osteoblast differentiation^141^. Hey1, which is involved in Notch signaling, can also be expressed by BMP2 signaling (Table 1)^142^. Particularly noteworthy is that kisspeptin-10 (KP-10), acting as a ligand for GPR54, has previously been identified to initiate the binding of NFATc4 to the BMP2 promoter via its interaction with GPR54. As a result of this interaction, BMP2 upregulates the expression of osteogenic genes by phosphorylating Smad1/5/9, thereby stimulating osteoblast differentiation in vitro^143^. Subsequent research unveiled that KP-10 encourages osteogenic differentiation of osteoblast progenitors and inhibits bone resorption in human cultured cells, while its administration to healthy male subjects resulted in a notable increase in the bone formation marker osteocalcin without corresponding effects on resorption markers^144^. Overall, the regulation of BMP2 activity and its signaling pathways represent a potential therapeutic target for conditions such as osteoporosis and other pathologically similar disorders.

Ubiquitin-mediated proteasome degradation also affects BMP signaling, with Smurf proteins regulating the interaction between osteoblasts and osteoclasts^145,146^. When Smurf1 is deficient, MEKK2 builds up, which triggers JNK activation, a necessary and sufficient event for osteocyte BMP sensitization^146,147^. Studies have demonstrated that Smurf2-deficient mice exhibit severe osteoporosis^148^. Osteoblasts deficient in Smurf2 exhibit elevated expression of RANKL. This phenomenon is attributed to Smurf2’s regulation of Smad3’s ubiquitination status and its disruption of the interaction between Smad3 and vitamin D receptors, ultimately resulting in alterations in RANKL expression^148^.

### Hedgehog signaling

#### Indian Hedgehog (Ihh)

Hh signaling plays an important role in cell proliferation and differentiation, and there are three Hedgehog homologs known to exist in mammals: Sonic Hedgehog (Shh), (Ihh), and Desert Hedgehog (Dhh). In cells gathered from mutant mice lacking smoothened (Smo), an Hh signal, osteoblastic differentiation is impossible^149^. As such, it is clear that in vitro mesenchymal and bone-forming cells rely heavily on the activity of Smo and the Hh signaling pathway (Fig. 2)^149^. Previous studies have demonstrated that Ihh is important in chondrocyte differentiation^150,151^. Ihh is produced by hypertrophic pre-chondrocytes, a group of cells in the inner perichondrium where osteoblast progenitors first appear^149,152^. The close proximity of these cell types shows the significant and interdependent effect of Ihh signaling on both osteoblast and chondrocyte differentiation^153^. In vitro experiments have demonstrated that osteoblast differentiation is compromised in mice lacking Ihh, and there is a deficiency in Runx2 expression. These findings suggest that Ihh signaling is capable of initiating osteoblastogenesis^154^. Additionally, a variety of mesenchymal and bone-forming cells have been stimulated in vitro to develop into osteoblasts by Ihh^155,156^.

In vitro studies have revealed that Speckle-type POZ protein (Spop), a component of the Cullin-3 (Cul3) ubiquitin ligase complex, positively regulates Hedgehog signaling^157,158^. Spop-deficient mutant mice exhibited defects in chondrocyte and osteoblast differentiation^158^. Parathyroid hormone-like peptide (PTHLH) expression was diminished in Spop mutants, while GLI family zinc finger 3 (Gli3) repressor form expression was up-regulated and GLI family zinc finger 2 (Gli2) expression remained unchanged, demonstrating that the Hh signaling was impaired^159^. Consistent with this finding, the Spop mutant’s skeletal defects were greatly ameliorated by decreasing Gli3 dosage^159^. A reduction in Gli3 dosage averted the formation of brachydactyly and osteopenia caused by Spop loss^159^. Therefore, Spop is an important positive regulator of Ihh signaling and skeletal development^159^.

#### Shh

The association between focal adhesion kinase (FAK) Tyr (397) expression and Sonic Hedgehog (Shh) expression suggests a potential link between FAK-regulated Osterix and the early differentiation of osteoblasts^160^. However, to better understand how FAK is controlled at the end of osteoblast differentiation, more research is needed. Purmorphamine, an Hh agonist, is a 2,6,9-trisubstituted purine that targets Smo transmembrane proteins^161^. The expression of Hh mediators such as Smo, patched 1 (PTCH1), Gli1, and Gli2 can be increased by purmorphamine (Fig. 2). Runx2 and BMP expression is also increased after Hh is activated by purmorphamine^162^.

From a clinical perspective, Shh has been implicated in the survival of tumor metastases. Research has suggested that signal peptide-CUB domain-EGF-related 2 (SCUBE2) operates in an autocrine fashion on tumor cells, releasing Shh that remains bound to the cell membrane^163–166^. This mechanism initiates the activation of Hedgehog signaling, consequently prompting the differentiation of osteogenic cells^166^. Notably, osteoblasts play a role in this mechanism by secreting collagen, which, in turn, activates the inhibitory leukocyte-associated immunoglobulin-like receptor 1 (LAIR1) signal in natural killer (NK) cells^166^. This cascade ultimately results in immune suppression and promotes the survival of tumor cells within the bone^166^.

#### Regulation of Hedgehog signaling

Pregnane X receptor (PXR) inhibits Hh signaling inducer genes like Gli1 and hedgehog-interacting protein (Hhip) while also inducing the expression of Hh signaling suppressor genes like cell adhesion associated (CDON), BOC cell adhesion associated (BOC), and growth arrest-specific 1 (GAS1)^167^. After treatment with Smo agonists, osteoblast differentiation and Gli-mediated transcriptional activity was significantly restored when Smo-mediated signaling was activated in these cells^167^. During osteoblast differentiation, Hh signaling significantly increases insulin-like growth factor 2 (IGF2) expression, which triggers the mTORC2-AKT signaling cascade. In turn, IGF2-AKT signaling stabilizes full-length Gli2, thereby enhancing the output of Hh transcriptional activation^168,169^.

In osteoblasts, the transmembrane protein SLIT and NTRK-like protein-5 (Slitrk5) are negative regulators of Hh signaling with a few known functions^170^. Osteoblasts exhibit a distinctive expression pattern of Slitrk5, where overexpression of Slitrk5 in osteoblasts impedes the activation of targeted genes involved in Hh signaling. Conversely, Slitrk5 deficiency in vitro enhances Hh signaling^171^. Slitrk5 has an extracellular domain that binds to hedgehog ligands and an intracellular domain that interacts with PTCH1^171^. Through binding with hedgehog ligands and PTCH1, Slitrk5 can inhibit Hh signaling activation, thus reducing osteoblastic differentiation.

#### Interaction between Hh signaling and Wnt signaling

The interplay between Hh signaling and Wnt signaling is intricate. Previous research has demonstrated that in Ihh-knockout embryos, β-catenin is not translocated into the nucleus and that the Wnt canonical signaling target genes are not expressed^88^. Thus, the Wnt signaling pathway can only be activated, in part, due to the presence of Hh signaling. Hh signaling also affects how Wnt9a and Wnt7b are expressed, where a noted decrease in their expression accompanies Ihh knockout^88^. Alternatively, Hh and Wnt signaling pathways could have intracellular crosstalk through common regulators, such as the fusion repressor^172^ and GSK3^173,174^. Some studies have demonstrated that BMP is important for Hh-actuated osteoblast differentiation, in addition to Hh’s interaction with the Wnt signaling pathway^175,176^. For example, during the development of long bones, Ihh and BMP signals are jointly regulated to promote the differentiation of osteoblasts^149^. Therefore, studying the interaction between Hh signaling and other signaling pathways could provide a new understanding of the regulatory process behind osteoblast differentiation.

### FGF signaling

#### Overview of FGF signaling

Fibroblast growth factors (FGFs) are a series of secreted peptides that control many developmental processes and are essential for controlling endochondral and intramembranous ossification (Fig. 2)^177^. For instance, craniosynostosis — a condition that presents with the early onset of osseous occlusion of the cranial suture — is usually acquired via mutations in FGF receptors 1-3^178,179^ (Fig. 2). During growth plate development, both FGF receptor 1 (Fgfr1) and FGF receptor 2 (Fgfr2) are expressed in the condensing stroma, contributing to cartilage formation^180^. Reserve chondrocytes express Fgfr2, and proliferating chondrocytes down-regulate it, while hypertrophic chondrocytes express Fgfr1^180^. At the final developmental stages, the tissues that produce osteoblasts and cortical bone, the perichondrium and periosteum, express both Fgfr1 and Fgfr2^180^. Osteoprogenitor cells typically use Fgfr1 signaling to encourage differentiation, but matured osteoblasts use it to prevent further differentiation^180^. As a result, Fgfr1 signaling influences osteoblast maturation in a stage-specific manner^180^. Unlike Fgfr1/2, Fgfr3 significantly regulates proliferating chondrocytes’ growth and differentiation^181^ while also mediating cortical thickness and bone mineral density in differentiated osteoblasts^182,183^. As such, human craniosynostosis and achondroplasia syndromes are primarily caused by Fgfrs mutations^177,184,185^. Additionally, exogenous FGF7 stimulates embryonic stem cell (ESC) differentiation by activating ERK-Runx2 signaling but does not affect the proliferation of osteoblasts^186^. FGF8 can stimulate Connexin 43 (Cx43) expression, which mainly occurs in osteoblasts and regulates osteoblast proliferation and differentiation^187^. Therefore, by controlling Cx43 expression, FGF8 can influence osteoblast differentiation. Furthermore, research indicates that FGF18 can also positively regulate osteoblast differentiation by mediating Fgfr1 and Fgfr2 activation via ERK1/2 and PI3K (Fig. 2)^188,189^.

#### Role of FGF signaling in osteoblast differentiation

Through phosphorylation of the MAPK signaling pathway, FGF2 can stimulate Runx2 expression, indicating that FGF2 participates in osteoblast differentiation^190^. Additionally, FGF2 can regulate the Wnt signaling pathway’s activity. Expressions of Wnt10b, LRP6, and β-catenin were reduced in FGF2-deficient BMSCs in vitro, and the inactivated GSK3β was also significantly reduced, suggesting that β-catenin degradation is possible^191^. By directly or indirectly controlling GSK3’s activity to regulate β-catenin stability, FGF2 may influence the Wnt signaling^191^.

The differentiation of osteoblasts also depends on FGFR. For example, Fgfr2-deficient mice exhibit a phenotype of decreased bone mineral density^192^. Different ligands of Fgfr2 include FGF7 and 10 (which are capable of activating FGFR2b) and FGF2, 4, 6, 8, 9 (which are capable of activating Fgfr2c)^193,194^. Adult Fgfr3-deficient mice displayed osteopenia, indicating that Fgfr3 also participates in osteoblast differentiation^182^. FGF18 can act as a physiological ligand for Fgfr3 to co-regulate osteoblast differentiation^195^. Furthermore, FGF18^–/–^ mice exhibited diminished endochondral and intramembranous bone formation, indicating that FGF18 can promote osteoblast differentiation independently of Fgfr3^195^. Moreover, FGF signaling has been implicated in cranial development, as mutations in FGFR2 have been linked to the autosomal dominant condition Crouzon syndrome. Mouse strains harboring the FGFR2 mutation (p.Cys342Arg) have demonstrated an enhancement in the osteogenic differentiation of MC3T3-E1 cells. This enhancement is achieved through the upregulation of the AMP-activated protein kinase (AMPK)-Erk1/2 signaling pathway^196^. Furthermore, the FGFR2 p.Cys342Arg mutation increased oxidative phosphorylation and altered mitochondrial dynamics from fusion to fission in MC3T3-E1 cells. This shift facilitated osteogenic differentiation and contributed to craniosynostosis in Crouzon syndrome^196^. A recent study showed that the stability of FGFR2 is maintained by OTU Deubiquitinase, Ubiquitin Aldehyde Binding 1 (OTUB1)^197^. OTUB1 diminishes the E3 ligase activity of Smurf1, which mediates FGFR2 ubiquitination, by hindering the binding of Smurf1 to E2^197^. When OTUB1 is absent, Smurf1 excessively ubiquitinates FGFR2, leading to its degradation in the lysosomes^197^. These discoveries suggest that OTUB1 actively participates in regulating osteogenic differentiation and mineralization in bone homeostasis by modulating the stability of FGFR2. This highlights OTUB1 as a potential therapeutic target for mitigating osteoporosis^197^.

Hereditary hypophosphatemic disorders are also associated with FGF signaling pathways, stemming from excess FGF23 — a phosphate (Pi)-regulating hormone produced by bone — which results in impaired skeletal growth and osteomalacia. A recent study explored the impact of Pi repletion and bone-specific deletion of FGF23 on bone and mineral metabolism in the dentin matrix acidic phosphoprotein 1 (Dmp1) knockout mouse model of autosomal recessive hypophosphatemic rickets (ARHR)^198^. The bone defects observed in Dmp1 knockout mice are only partially attributable to FGF23-induced hypophosphatemia^198^. The study proposed that simultaneous restoration of DMP1 levels and inhibition of FGF23 could effectively rectify mineral and bone disorders associated with ARHR. Moreover, furin demonstrated the ability to cleave FGF23 in vitro, suggesting a potential therapeutic avenue^198^. Indeed, inactivation of furin in osteoblasts and osteocytes increased circulating intact FGF23 by 25%^199^ without significantly impacting serum phosphate levels. Therefore, therapeutically targeting excess FGF23 in patients suffering from hereditary hypophosphatemic disorders could represent a novel treatment modality.

#### Regulation of FGF signaling

Engrailed homeobox 1 (En1) can regulate FGFR-mediated signaling. ERK activation is inhibited when En1 is mutated, and the ERK activation is confined to mature intracranial osteoblasts of the wild-type skull^200^. The outer periosteal osteoblasts affected by En1 ablation lost the FGF target gene sprouty RTK signaling antagonist 2 (SPRY2)^200^. P38, MAPK, and PKC, the effectors of FGF signaling known to influence osteoblast differentiation, may also be influenced by En1^200–202^. En1 and FGF’s coordination of osteoblast differentiation will be better understood if these regulatory pathways are precisely described in a spatiotemporal manner.

The Sprouty family functions as an inhibitor of FGF signaling (Fig. 2). Basic FGF stimulation causes Sprouty2 expression to rise to high levels^203^. The increased presence of Sprouty2 directs ERK1/2 phosphorylation after basal FGF activation and Smad1/5/8 after BMP activation (Fig. 2)^203^. Expressions of Ocn, ALP, and Osterix mRNA were all inhibited by Sprouty2. Additionally, osteoblast matrix mineralization was stifled by Sprouty2. By inhibiting the expression of markers in differentiated osteoblasts, like Runx2 and ALP, and reducing FGF-ERK1/2 and BMP-Smad signaling, osteoblast differentiation and proliferation are negatively regulated by Sprouty2^203^.

## Noncanonical signaling pathways in osteoblast differentiation

### Ephrin signaling

#### Overview of Ephrin signaling

Ephrins have bidirectional signal transduction capabilities. This class of signaling proteins falls into two categories: class A (ephrins A1 through A5), which bind to GPI-anchored EphA receptors (A1 through A10), and class B (ephrins B1 through B3), which bind to EphB tyrosine kinase receptors B1 through B6^204^. The interaction between Ephrin B and EphB, a transmembrane protein with a cytoplasmic domain, facilitate bidirectional signaling in cells. EphrinB2 from osteoclasts and EphB4 from osteoblasts, for instance, combine to create the signal between the two cells^205^. EphrinB2 on the surface of osteoclasts mediates EphB4 activation and promotes osteoblast differentiation^206^ (Fig. 1). In contrast, activation of EphrinB2-fold EphB4 on the surface of osteoclasts inhibits C-Fos/NFATc1 signaling of osteoclasts, thereby preventing osteoclast differentiation^206^. EphB4 signaling can prompt osteoblasts to express transcription factors like DLX5, Osx, and Runx2, underscoring the significance of Ephrin signaling in osteoblast differentiation^25^. Additionally, the inactivation of ras homolog family member A (RhoA) in osteoblasts may be required for Ephrin signaling to promote osteoblast differentiation^207^ (Fig. 3), with a study finding that suppression of osteoblast differentiation by EphrinA2(EfnA2)-EphA2 is mediated by increased RhoA activity^208^.

#### Ephrin signaling regulation

The effects of stage-specific EphrinB2 deletion on bone strength vary. The early loss of EphrinB2 in osteoblasts results in osteoblast apoptosis, delayed onset of mineralization, and increased bone flexibility. Subsequent deletion of EphrinB2 in osteocytes targeted by the cell can lead to a fragile bone phenotype and heightened osteocyte autophagy^209^. When drugs were administered to cultured osteoblasts to prevent EphrinB2 from interacting with EphB4, the final stages of osteoblast differentiation and mineralization were repressed^210^. When inhibiting the interaction of EphrinB2 with EphB4 in vivo, early-stage osteoblast numbers increased while late-stage osteoblast numbers decreased, suggesting the existence of an EphrinB2:EphB4-dependent checkpoint in the process of osteoblast differentiation^210^. When targeting this EphrinB2:EphB4 checkpoint, the final osteoblast differentiation is blocked, interrupting the initiation of mineralization^210^. Therefore, these studies suggest that this crucial checkpoint controls the onset of bone mineralization during the late stage of osteoblast differentiation.

Additionally, in osteoblast lineages, the interaction between EphrinB1 and EphB2 impacts the expression from Runx2 to Osx, whereas the interaction between EphrinB2 and EphB4 allows for ALPl expression^210^. The interaction between EphrinB1 and EphB2 improves their function in osteoblast differentiation and bone formation^211^. EphrinB1 is necessary for early osteoblast marker expression like Osx and Runx2, with TAZ — a transcriptional coactivator with a PDZ-binding motif — causing Osx expression when translocated into the nucleus through stimulating EphrinB1 reverse signaling with aggregated EphB2^211^. In the osteoblast lineage, the interaction between EphB2 and EphB4 not only facilitates the differentiation of late osteoblasts but also prevents the formation of osteoclasts^211^.

The TGF-β1-Scx-EfnA2 axis can negatively regulate periodontal ligament (PDL) tension-induced osteoblast differentiation. TGF-β1-Smad3 signaling and EfnA2 are upstream and downstream regulators of scleraxis (Scx) in PDL cell response to tension, respectively^212^. Previous work has shown that Scx knockdown completely inhibits tension-induced EfnA2 expression, suggesting that tension-induced Scx inhibits PDL osteoblast differentiation by inducing EfnA2 expression^212^. This example of the co-regulation of osteoblast differentiation by coordinating the TGF-β and Hh signaling pathways exemplifies the importance of studying the intersection of various signaling pathways of osteoblast differentiation.

### TLR/NF-κB signaling

#### Overview of TLR/NF-κB signaling

RelA (p65), RelB, cRel, NF-κB1 (P50), and NF-κB (P52) form the components of NF-κB signaling, usually kept sequestered by κB inhibitors within the cytoplasm^213^ (Fig. 3). During bone repair, inflammation is common and has been shown to prevent bone regeneration^214^. Osteoblast differentiation was inhibited by NF-κB signaling stimulation in previous research^215^. Cytokines such as tumor necrosis factor (TNF), interleukin-1 (IL-1), interleukin-6 (IL-6), and interleukin-7 (IL-7) facilitate NF-κB activation and hinder osteoblast function in osteoporosis^213^. Furthermore, TNF-α and IL-1 can regulate osteoblast differentiation by down-regulating the promoter function of osteocalcin, a key gene in osteoblasts^214,216^. The expression of those cytokines is elevated when NF-κB signaling is stimulated, which will inhibit osteoblast differentiation^217^.

#### TLR/NF-κB signaling regulation

IKK-NF-κB stimulation promotes β-catenin ubiquitination and degradation through Smurf1 and Smurf2, inhibiting the Wnt signaling pathway and stimulating Runx2 degradation (Fig. 3). Furthermore, TNF can induce p65 to bind to Smurf1 and Smurf2 promoters in MSCs^215^. Therefore, TNF may hinder osteoblast differentiation by activating NF-κB signaling. In the healing of occlusion injuries in rodents, the levels of β-catenin and Runx2 decreased alongside increased expression of p65 and IκBα^215^. However, after hindering IKK-NF-κB signaling, there was a significant increase in the expression of β-catenin, OCN, and Runx2^215^. Thus, IKK-NF-κB activation is involved in the degradation of β-catenin, ultimately preventing osteoblast differentiation and bone formation^215^. Similarly, melatonin was found to counteract the activation of the NF-κB pathway by inhibiting TNF-α, thereby reducing osteogenic differentiation and inflammation in bone marrow-derived mesenchymal stem cells (BMSCs)^218^.

Rocaglamide-A, a suppressor of NF-κB signaling, inhibits phosphorylation of NF-κB to promote osteoblast differentiation^219^. Conversely, p65 overexpression prevents the promotion of osteoblast differentiation by rocaglamide-A^219^. Rocaglamide-A inhibits p65 protein phosphorylation and the accumulation of p65 in the nucleus, reducing the transcriptional activity for NF-κB^219^. According to this work, the NF-κB p65 protein is engaged with the enactment of the NF-κB signaling, and recaglamide-A can forestall the NF-κB inhibitory impact on osteoblast differentiation by preventing the phosphorylation of p65^219^.

Osteoblasts’ autophagy-related function changes significantly when the NF-κB signaling pathway is disabled, demonstrating a possible connection between the two mechanisms^220^. However, according to some studies, NF-κB appears to play two roles in autophagy. NF-κB activation has the potential to encourage autophagy in some kinds of cells, such as myocardial cells^221^, and can also hinder autophagy in other cells like porcine granulosa cells^220,222^. Therefore, the mechanism of NF-κB in the regulation of autophagy during osteoblast differentiation still needs further research.

Toll-like receptor 4 (TLR4) is also a key factor in NF-κB signaling, with osteoblast expression of TLR4 necessary to activate the NGF-TrkA signaling required for stress-induced bone formation^223,224^. For example, in TLR4-conditioned knockout mice with normal adult bone mass and strength, loss of TLR4 signaling significantly reduced lamellar bone formation after stress loading^225^. Inhibition of TLR4 signaling decreased the expression of Ngf in primary osteoblasts^225^. Bone RNA sequencing in TLR4-conditioned knockout mice and wild-type pups revealed dysregulated inflammatory signaling three days after mechanical osteogenic loading, revealing the important role of osteoblast TLR4 in bone adaptation to mechanical forces^225^.

### Notch signaling

Notch is cleaved by ADAM, a disintegrin and γ-secretase complex containing presenilin 1 or 2 that is formed by Notch ligand interactions^226^. Upon cleavage, the cytoplasmic Notch intracellular domain (NICD) is liberated and translocated to the nucleus (Figs. 3, 4)^226^. There, it forms a complex that controls gene transcription via binding with CSL family members^226^. This complex can activate HEY1 expression, with HEY1 then binding to Runx2 to prevent osteoblast differentiation (Fig. 4)^227^. NICD can block NFAT signaling by interacting with Foxo1, which also results in the inhibition of osteoblast generation. NICD also impedes Wnt/β-catenin signaling^228^. Hes1 is one of the downstream target genes of the Notch signaling pathway^228,229^ (Table 1). Hes1 silencing in vitro suggests that NICD inhibits Wnt/β-catenin signaling primarily through Hes1, where the binding of Hes1 to LEF-1 or transducin-like enhancer protein (TLE) may inhibit Wnt signaling^228,229^.

Notch signaling exhibits a bidirectional effect on osteoblast differentiation, as it can either inhibit or promote osteoblast differentiation depending on the stage of osteoblast differentiation and the timing of Notch activation^230^. Studies have shown that activation of Notch signaling can promote the mineralization process of osteoblasts^231,232^, while it has also been shown that overexpression of Notch1 can inhibit osteoblast differentiation by inhibiting the Wnt/β-catenin signaling pathway^233^. It was shown that Notch1 not only inhibits terminal osteoblast maturation but also promotes immature osteoblast formation^234^. Moreover, it was observed that Notch signaling enhances the effectiveness of BMP9-induced BMP/Smad signaling, leading to an upregulation in the gene expression of critical osteogenic factors induced by BMP9 in MSCs, such as Runx2, Colla1, and inhibitor of differentiation^234^.

Forkhead box protein O1 (Foxo1) inhibits osteoblast production through Notch signaling. Foxo1 forms complexes with NICD and Mastermind to inhibit gene expression^235,236^ (Figs. 3, 4). As an illustration, Foxo1 inhibits nuclear factor of activated T cells (NFAT) signaling in activated T cells. NFATc1 and NFATc2, in turn, promote the production of osteoblasts, bone formation, and osteoclasts^235^. Through its interaction with Foxo1, Notch directly and indirectly inhibits the generation of osteoblasts and osteoclasts^236^. GSK3 can phosphorylate NFATc, resulting in its nuclear export^237^. The co-localization of Notch2’s NICD with GSK3 in the nucleus can inhibit Wnt/β-catenin signaling^235^ and suppress osteoblast differentiation. The spalt-like transcription factor 4 (SALL4) can also regulate osteoblast differentiation by inhibiting Notch2’s ability to translocate into the nucleus^238^.

Epigenetic molecular processes dynamically modify both DNA and histone tails, influencing the spatial organization of chromatin and fine-tuning the outcome of Notch1 transcriptional response^239^. Although researchers have examined the interaction between histone deacetylase 1 (HDAC1) and Notch in vitro and in *Drosophila* wing development, the precise role of this interaction in mammalian skeletal development and disorders remains uncertain^240,241^. In a murine model of osteosclerosis, HDAC1/2 has been identified as a contributor to the disease pathogenesis, which has been attributed to the conditionally cre-activated expression of the Notch1 intracellular domain in immature osteoblasts^242^. Significantly, targeted homozygous deletions of HDAC1/2 in osteoblasts result in partial alleviation of osteosclerotic phenotypes^242^. When HDAC1/2 was specifically deleted in osteoblasts of male and female mice, there was an absence of overt bone phenotypes, even in the absence of the Notch1 gain-of-function allele^242^. These findings provide evidence supporting the idea that HDAC1/2 contributes to the pathogenic signaling of Notch1 in the mammalian skeleton^242^.

### Hippo signaling

Cell proliferation, apoptosis, and stem cell development rely heavily on the Hippo signaling cascade. When Hippo signaling is at an “on” state, YAP and TAZ undergo phosphorylation and are inhibited downstream^243^. YAP/TAZ are broken down in the cytoplasm, either through interaction with 14-3-3 proteins or through degradation mediated by the proteasome (Fig. 3)^243^. Yet, when Hippo signaling is inactive, YAP/TAZ translocates into the nucleus where they bind to the TEA domain transcription factor (TEAD), jointly regulating gene expression (Fig. 3)^244^. By initiating the downstream RhoGTPase-actomyosin signaling cascade, matrix metallopeptidase 14 (MMP14) can trigger β1-integrin activation and thereby promote YAP/TAZ nuclear transfer to regulate gene expression^245^.

YAP is an important component within Hippo signaling (Table 1). It regulates cell proliferation and apoptosis via cooperating with the Tead/Tef family and activating target genes in the Hippo signaling pathway^246^ (Fig. 3). In vitro experiments have exhibited that YAP can inhibit bone marrow MSC osteogenesis, subsequently reducing the rate of osteoblast differentiation^247^. According to previous research, YAP is a mediator of the Src/Yes tyrosine kinase pathway, which can interact with Runx2 to suppress Runx2 transcriptional activity to inhibit osteocalcin expression^247^.

YAP’s nuclear localization is influenced by numerous factors, which, in turn, limit its transcriptional activity. For instance, the MAPK signaling pathway’s expression of ERK and JNK controls the degree of YAP phosphorylation^248^. However, YAP phosphorylation can be accelerated by Src/Yes kinase^249^. Conversely, the Runx2-YAP complex separates when Src/Yes kinase is inhibited^249^. By blocking the interaction between YAP and 14-3-3 protein through YAP phosphorylation, which is mediated by AKT kinase, YAP can remain in the cytoplasm and not be degraded, ultimately preventing the expression of osteoblast-related genes^250^.

Transformation-associated protein 53 (Trp53) is a key regulator that regulates the activation of TAZ to regulate the osteoblast differentiation^251^. Elimination of Trp53 in osteoblast lineages markedly boosts osteogenesis, bone formation, and bone remodeling^252^. The lack of Trp53 significantly increases the transcriptional activity of TAZ by hindering TAZ phosphorylation and its translocation into the nucleus, while this activity is notably reduced upon enforced expression of Trp53^251^. Trp53 is linked to TAZ and reduces the stability of the TAZ protein, promoting its degradation through ubiquitination mediated by beta-transducin repeats-containing proteins (β-TRCP)^251^. To sum up, Trp53 governs chondrogenesis and intrachondral ossification by inhibiting TAZ activity and stability. This implies that manipulating Trp53 signaling might offer a promising avenue for addressing fracture healing, ectopic ossification, arthritis, and various other bone-related disorders^251^.

Hippo signaling also crosstalks with other signaling pathways, including the Wnt and TGF-β pathways, to modulate osteoblast differentiation. Hippo signaling stimulates TAZ to cooperate with disheveled (DVL) in the cytoplasm, which limits Wnt/β-catenin signaling^253^. By preventing DVL phosphorylation, TAZ can block Wnt/β-catenin signaling^253^. Similarly, the Hippo signaling pathway inhibits TGF-β signaling by preventing Smad complexes like Smad2/3 from translocating into the nucleus through the cytoplasmic localization of TAZ/YAP^254,255^. Clinically, the TAZ/YAP signaling pathway has recently been linked to abnormal differentiation of mesenchymal lineage (MLin) cells, which leads to the formation of bone within soft tissues of the musculoskeletal system following traumatic injury^256^. Recent studies have demonstrated that discoidin domain receptor 2 (DDR2) is significantly and specifically upregulated in collagen-expressing heterotopic ossification MLin cells^256^. DDR2 acts as a cell surface receptor for fibrillar collagen and serves as a crucial regulator of MLin cell function in heterotopic ossification formation^256^. Mechanistically, perturbation of DDR2 alters focal adhesion orientation and subsequent matrix organization, thereby modulating the YAP/TAZ-mediated signaling of MLin cells through focal adhesion kinase FAK and YAP/TAZ^256^. Therefore, the interactions between ECM and DDR2 play a crucial role in driving heterotopic ossification, presenting a potential novel therapeutic target for addressing this pathological condition^256^.

### Piezo1 and Piezo2 signaling

Bone tissue responds to mechanical stress stimulation, with both unloading and loading of mechanical stress exerting significant effects on osteoclast differentiation and function, as well as osteoblast differentiation and function^257^. The bone microenvironment and metabolic activity is influenced by mechanical stress^257^. At present, the understanding of mechanoreceptors is thought to involve ion channels, extracellular matrix adhesion molecules, and the cytoskeleton, yet the current understanding of these receptors is incomplete^258^. Piezo1 and Piezo2 are important mechanosensitive channels. Piezo1 and Piezo2 have been identified as crucial players in regulating osteoblast differentiation and function^259–261^. Deletion of Piezo1 or Piezo2 in osteoblast or osteoclast lineage cells causes a severe osteoporosis phenotype with numerous spontaneous fractures, specifically in osteoblast lineage cells, indicating that Piezo1 and Piezo2 are important in bone formation and bone function^262^.

Piezo1 and Piezo2 mechanosensitive channels have been recognized as critical force sensors essential for bone development and osteoblast differentiation^263^. Depletion of Piezo1, and particularly Piezo1/2, in mesenchymal or osteoblast progenitor cells results in spontaneous bone fractures in newborn mice^263^. This outcome was attributed to the suppression of osteoblast differentiation and increased bone resorption^263^. Furthermore, the absence of Piezo1/2 conferred resistance to additional bone loss induced by unloading in both bone development and homeostasis^263^. Mechanistically, Piezo1/2 channels transmit signals from fluid shear stress and extracellular matrix stiffness, triggering calcium influx that activates Calcineurin^263^. This activation leads to the coordinated activation of NFATc1, YAP1, and β-catenin by inducing their dephosphorylation, along with the formation of NFAT/YAP1/β-catenin complexes (Fig. 3)^263^. Piezo1 and Piezo1/2 mutant mouse bones have reduced Yap1 and β-catenin activities, and enhanced β-catenin activity partially rescues the phenotype^263^.

Zou et al. discovered that PIEZO1 functions in osteoblast lineage cells to regulate bone remodeling by sensing mechanical loading^264^. Their research demonstrated that PIEZO1 regulates YAP signaling, which in turn controls the expression of various bone matrix proteins, including several types of collagens (Fig. 3)^264^. These collagens’ expression is contingent upon a PIEZO1-dependent response to mechanical stimulation^264^. This suggests that PIEZO1 may also play a role in regulating bone mass and bone strength in humans in vivo^265^.

For bone growth to occur, a specific type of highly angiogenic blood vessels known as type H vessels is required. These vessels provide a pathway for osteoblasts surrounding them^266^. Towards the end of adolescence, type H vessels undergo a transition to a quiescent type L endothelium, losing their ability to support bone growth^267,268^. Dzamukova et al. discovered that mechanical forces, linked to increased body weight at the end of adolescence, activate the mechanoreceptor PIEZO1, leading to heightened production of FAM20C (Golgi-associated secretory pathway kinase) in osteoblasts^266^. FAM20C, the primary kinase of the secreted phosphoproteome, phosphorylates DMP1, previously recognized as a crucial factor in bone mineralization^269,270^. Following this, osteoblasts release dentin matrix protein 1 in a pulsatile fashion^266^. Extracellular DMP1 inhibits vascular endothelial growth factor (VEGF) signaling by preventing the phosphorylation of vascular endothelial growth factor receptor 2 (VEGFR2)^266^. This process results in the conversion of type H vessels into type L vessels, decreasing bone growth activity and fostering heightened bone mineralization^266^. Consequently, targeting PIEZO1 may offer a novel approach for addressing bone-related diseases^266^.

### Hormone regulation

Hormones also play significant role in regulating osteoblast differentiation. Thyroid-stimulating hormone (TSH) is an important hormone that can act on the thyroid gland to produce two thyroid hormones, thyroxine (T4) and tri-iodothyronine (T3)^271^. A study found that a novel form of the TSHβ subunit, TSHβv, can promote osteoblast differentiation^272^.

The sympathetic nervous system boosts bone breakdown by increasing Rankl in early bone cell precursors, a process needing ATF4 phosphorylation. In mice lacking Adrb2 and gonads, bone breakdown decreases, whereas in hypogonadal mice with low sympathetic activity, bone breakdown increases. This discrepancy might be due to CART, a neuropeptide influenced by leptin, which regulates RankL to curb bone breakdown. Leptin-driven neural pathways manage two aspects of bone remodeling^273,274^.

Insulin signaling can regulate osteoblast differentiation^2,275,276^ (Fig. 3). Study found that insulin signaling can inhibit Twist2 to regulate osteoblast differentiation^277^. Twist2 is one of the inhibitor of Runx2^276^ (Fig. 3). The transcription factor forkhead box O1 (FoxO1) acts as an inhibitor of insulin signaling. Once Ocn is synthesized, it undergoes γ-carboxylation, followed by export and storage bound to the mineralized bone extracellular matrix (ECM). The inactivation of Ocn can be hindered by the cellular import of glutamate, which inhibits the activity of the γ-carboxylase enzyme^278^ (Fig. 3). Alternatively, γ-carboxylated Ocn bound to the ECM can undergo partial decarboxylation and be released into the general circulation through bone resorption^2,278^. Besides, Ocn could activate β-cells, and the activated β-cells can promote insulin signaling to promote osteoblast differentiation.

## Osteoblast transcriptional factors

Several homeodomain proteins are included in the group of transcription factors that control osteoblasts: Smads, CCAAT/enhancer binding proteins β (C/EBβ), C/EBPδ (Table 1), lymphatic enhancer factor (Wnt effector), Twist, Runx1/Cbfβ, activating transcription factor 4 (ATF4), Runx2, and Osterix, of which the latter four are the major transcription factors that function within osteoblast differentiation (Fig. 4; Table 1).

Transcriptional modulators that function as “master switches” coordinate the transformation of MSCs into tissue-specific cell types. Runx2 is pivotal in bone formation^279^. During differentiation, it plays a major role in regulating cell growth and the activation or repression of genes^279^. Even though osteoblast differentiation mainly relies on Runx2 and Osterix, other important genes like Runx1 and Cbfβ are also required for differentiation (Fig. 4)^3^.

### Runx2

Runx2, also known as Cbfa1 or PEBP2, is a member of the RUNT-related transcription factor family, which also includes Runx1 and Runx3^280^. Runx2 is particularly important for osteoblast differentiation. All Runx family proteins can bind to Cbfβ to form stable heterodimers with a DNA-recognition sequence of TGTGGT^281^. Runx2 has an activation domain that turns on the genes for osteocalcin and COL1A1 in addition to its conserved DNA-binding domain^280,282,283^. As a result, Runx2 is an early marker of osteoblast differentiation, which is indicated by the relative timing of its expression^284^ (Fig. 4). At the stage where MSCs first differentiate into osteoblast precursor cells before transforming into immature osteoblasts, Runx2 expression is upregulated^284^. However, by the final stages of osteoblast differentiation and formation, Runx2 expression gradually decreases (Fig. 4)^284,285^. Glucose, the main nutrient for osteoblasts, is transported into these cells through Glut1, which is expressed before Runx2. Glucose uptake facilitates osteoblast differentiation by suppressing AMPK-dependent proteasomal degradation of Runx2 and also enhances bone formation by inhibiting another aspect of AMPK function^286^.

Runx2 is abundant in bone and calcified cartilage but is also expressed in the thymus and testicles^287,288^. Runx2-deficient mice failed to form bone, indicating that Runx2 is necessary for the formation of both membranous and endochondral bone^280,289,290^. Osteoblast-specific genes were expressed when Runx2 was produced in cultured skin fibroblasts, suggesting that Runx2 could promote osteoblast differentiation^280^. Runx2 may govern the transition from the growth phase to the postproliferative phase by inhibiting progenitor cell proliferation, thus serving as a critical element in the development of cells within the osteogenic lineage^291^. Thus, Runx2 may induce the gene expression needed for lineage identification and mesenchymal differentiation in early bone progenitor cells.

Due to this critical role in bone homeostasis, Runx2 has also been implicated in the symptomatic progression of bone-associated cancers. A recent study found that increased expression of Runx2 is responsible for bone destruction found in multiple myeloma (MM)^292^. The paper demonstrated that Runx2 promotes the suppression of osteoblast activity and enhancement of osteoclast activity by multiple myeloma cells using in vitro and in vivo approaches^292^. Notably, the data suggests that therapeutic blockade of Runx2 could safeguard against bone degradation by preserving the equilibrium between osteoblast and osteoclast functions in MM^292^.

#### Co-activators of Runx2

Numerous transcriptional coactivators can engage with Runx2. Cbfβ is the most important of these factors because it can bind to Runx family proteins to form heterodimers, improving Runx2’s stability and subsequently facilitating Runx2 binding to target DNA sequences (Fig. 4). Cbfβ settles Runx2 by restraining ubiquitination, thus preventing Runx2 degradation and further promoting the expression of osteoblast-specific genes^293^. Cbfβ is also crucial in stimulating osteogenesis by inhibiting the expression of the adipogenesis regulatory gene C/EBPα and activating Wnt10b/β-catenin signaling^91^ (Table 1). Wnt10b expression was down-regulated in Cbfβ-deficient cells, and another analysis showed that Cbfβ increased the transcriptional expression of Wnt10b^91^. Furthermore, adipocyte development was suppressed in the presence of Wnt3L conditioned medium containing Wnt3a, but osteoblast differentiation was not reversed^91^. According to these findings, Runx/Cbfβ controls osteoblast differentiation independently of Wnt10b/β-catenin signaling and suppresses the transformation of MSCs and osteoblasts into adipocytes^91^.

In addition to Cbfβ, co-activators such as monocytic leukemia zinc finger protein (MOZ), MOZ-related factor (MORF), and others play a crucial role in facilitating Runx2’s control over osteoblastogenesis. MOZ and MORF belong to the MYST family that comprises histone acetyltransferases, which can interact with Runx2’s activation domain to increase osteocalcin expression^294^. Grg5, pRb, and TAZ are also effective Runx2 activators^295,296^ that can increase Runx2’s transcriptional activity through their interaction with the protein^297,298^. These co-activators interact with Runx2 to enhance the transcriptional activity of Runx2 and promote the regulation of Runx2 on osteoblast-specific gene expression, thus promoting osteoblast differentiation^297,298^.

Vestigial-like family member 4 (VGLL4) disrupts TEAD-mediated Runx2 transcriptional repression, thereby facilitating osteoblast differentiation and bone development (Table 2)^299^. TEAD transcription factors are inhibitors of Runx2 transcriptional activity, which can act independently of YAP binding, severely inhibiting osteoblast differentiation^299^. In addition, VGLL4 attenuates TEAD transcriptional repression by directly competing with Runx2 for binding of TEAD through its two TDU domains^299^. VGLL4 can remove the inhibitory function of TEADs by disrupting its interaction with Runx2^299^. Additionally, the absence of VGLL4 in MSCs has shown a skeletal defect similar to global VGLL4-deficient mice, along with the knockdown of TEAD or overexpression of Runx2 in VGLL4-mutant osteoblasts reversing osteoblast differentiation inhibition^299^. These results indicate that VGLL4 can control the Runx2-TEADS transcription complex to control osteoblast differentiation and bone formation^299^.

**Table 2:** Transcription factor partners of Runx2.

Co-activator	Role	Co-repressor	Role
**Cbfβ**	Enhances Runx protein binding to DNA Stabilizes Runx by inhibiting ubiquitination-mediated degradation	**Hey1**	Negatively regulates Runx2 by binding with Runx2 to suppress it
**SIRT1**	Interacts with Runx2 and promotes its trans-activation potential	**Smurf1/2**	Degrades Runx2 by ubiquitination-mediated proteasome degradation pathway
**TAZ**	Stimulates osteogenic differentiation via interacting with Runx2	**COUP-TFII**	Impairs Runx2-dependent osteocalcin promoter activation
**P300**	Positively regulates Runx2 by mediating Runx2 acetylation	**Grg/TLE**	Inhibits Runx2-dependent transcriptional activation of OCN genes
**VGLL4**	Breaks TEAD-mediated Runx2 transcriptional inhibition	**EGFR**	Increases the content of transcription corepressor HDAC4 and HDAC6 and inhibits the expression of Runx2

Runx2 can also promote gene expression by interacting with the myeloid Elf-1-like factor (MEF) and binding to an osteoblast markers’ promoter, like OPN, close to MEF (Table 2)^300^. The presence of these two transcription factor response elements (RRE and ERE) near the promoter’s proximal region is necessary for MEF and Runx2 to function on the promoter of bone marker genes^300^. When getting close to these two elements, MEF and Runx2 show significant synergistic effects on promoter activity. MEF and Runx2 synergistically affect OPN, OCN, and ALP promoters^300^.

Sirtuin 1 (SIRT1) has the potential to positively regulate Runx2 (Table 2). Runx2’s activity is enhanced by SIRT1, a Class III histone deacetylase^301^. In vitro experiments showed that Runx2 expression did not change when SIRT1 was absent, but osteoblast differentiation was inhibited, and Runx2 target protein expression was decreased^301^. By interacting with Runx2, SIRT1 can enhance its trans-activation activity^301^. As Osx is one of Runx2’s downstream targets, SIRT1 can trigger Runx2’s activation and promote Osx expression, which in turn targets osteoblast-specific gene expression and promotes osteoblast differentiation^301^.

#### Co-repressors of Runx2

One particularly important regulatory mechanism that is utilized to help govern the transcription of osteoblast-linked genes is protein degradation. As Runx2 is a critical transcription factor in promoting osteoblast differentiation, the degradation of this protein through the proteasome pathway has been shown to inhibit the rate of osteoblastogenesis^302^. Smad6 can initiate Smurf1-induced Runx2 degradation, triggered by the ubiquitin isopeptide ligase (E3) Smurf1^303^. E3 ligase can interact with Hsc/HSP70, which negatively regulates osteoblast differentiation by promoting Runx2 ubiquitination degradation^304^. Additionally, a novel AMPK pathway has been identified to modulate Runx2 degradation via ubiquitin degradation^305–307^. However, the extent to which Runx2, and any subsequent osteoblast differentiation, is altered by AMPK via ubiquitin degradation or rather is more significantly mediated by alternative mechanisms requires further investigation due to recent conflicting findings^307,308^.

The epidermal growth factor receptor (EGFR) signaling pathway plays an important role in tissue development and tumor development and has been previously shown to regulate the expression of Runx2 and Osterix (Table 2). EGFR diminished Runx2 and Osx expression and increased the expression of inhibitors HDAC4 and HDAC6 in differentiated osteoblasts^309^. In vitro overexpression of HDAC4 diminishes Runx2 levels because HDAC deacetylates lysine residues in histones, preventing transcriptional machinery from accessing DNA and repressing the transcription of target genes^309^. Subsequently, the mechanism of EGFR inhibiting Runx2 activity may be accomplished through the upregulation of HDAC4 and HDAC6 expression^309^.

Chicken ovalbumin upstream promoter-transcription factor II (COUP-TFII) is an orphan nuclear receptor belonging to the steroid thyroid hormone receptor superfamily. It physically interacts with Runx2^310^. Specifically, COUP-TFII suppresses Runx2-dependent osteocalcin promoter activation^310^. COUP-TFII inhibits Runx2 DNA binding to the osteocalcin gene. Conversely, Runx2 suppresses COUP-TFII expression by directly binding to the COUP-TFII promoter^310^. Hence, this indicates that COUP-TFII may negatively regulate osteoblast differentiation through its interaction with Runx2^310^.

### Runx1

Runx1, also called AML1 or Cbfa2, belongs to the RUNT-related transcription factor family. Runx1 and Cbfβ form a heterodimer and are highly expressed in osteoblasts and pre-osteoblasts^311^. Runx1 controls how HSCs become mature blood cells^312^ and is key in the development of pain-transmitting neurons^313^ (Fig. 1). Additionally, in vitro studies have proved that Runx1 is fundamental for early chondrocyte differentiation^314^ and osteoblast differentiation^315^. Runx1 positively regulates osteoblast lineage gene expression at various stages of differentiation, promoting bone formation and inhibiting adipogenesis (Fig. 1)^315^. Runx1 primarily participates in the early stage of osteoblast differentiation (Table 1). In our previous study, we observed that during this period, when bone marrow-derived mesenchymal stem cells (BMSCs) differentiate into chondrocytes and osteoblasts, Runx1 and Runx2 are co-expressed^314^. Runx1 stimulates chondrocyte commitment to the osteoblast lineage and augments bone formation by increasing both chondrogenesis and osteogenesis. Furthermore, overexpression of Runx1 in BMSCs initiates chondrocyte differentiation, while a deficiency in Runx1 results in reduced expression of Runx2 and impaired differentiation of both chondrocytes and osteoblasts^316^. Besides, previous study also found that loss of Runx1 will decrease the expression of SOX9, Ihh, PTC in the growth plate, while the expression of OCN, Osx, Runx2 and ATF4 are also decreased^315^. These results demonstrated that Runx1 is important in osteoblast differentiation and bone formation.

Runx1 is very important for coordinating several pathways implicated in bone formation and homeostasis, including BMP and Wnt/β-catenin signaling (Fig. 4). Our lab found that Runx1 plays a significant role in postnatal bone homeostasis by binding to BMP7, activin-like kinase-3 (ALK3), and ATF4 promoters to activate the corresponding genes^311^. Expression of these genes produces critical transcription factors in osteoblast differentiation, which, after activation, can further regulate osteoblast-specific gene expression to promote osteoblastogenesis.

In addition, our lab found that Runx1 regulates osteoblast-adipocyte lineage via inducing Wnt/β-catenin signaling and restraining adipogenic gene transcription^311^. Previous research in our lab has demonstrated that Wnt10b/β-catenin signaling is crucial to the composition of the osteoblast-adipocyte lineage^91^ (Fig. 4). In vitro research has previously established that β-catenin signaling is diminished with Runx1 deficiency^311^. When the Wnt/β-catenin signaling pathway is inhibited, osteoblast cell lines will differentiate into adipocytes^311^. Therefore, Runx1 can regulate the differentiation pathway of osteoblasts by controlling the expression of β-catenin in the Wnt signaling pathway.

Along with adipogenic gene regulation, Runx1 can directly interact with the Ihh promoter to mediate its expression, up-regulate the transfer of chondrocytes to osteoblasts, and promote the expression of chondrogenesis/osteogenesis-related genes^315^. Additionally, our lab found that Runx1 also mediates osteoblast differentiation and bone formation by binding to Runx2 and OCN gene promoters and multiple bone-specific genes, leading to increased Runx2 expression^317^. The Runx2 promoter’s high binding efficiency at binding site 4 is due to the acetylation of chromatin-associated histones due to Runx1’s interaction with histone acetyltransferase, which alters chromatin conformation and activates transcription^317^. Runx1 can partially replace the function of Runx2 and positively regulate osteoblast differentiation^311^. Overexpression of Runx1 in Runx2-deficient osteoblasts can salvage the expression of key regulatory proteins (ATF4 and OCN), rescue bone formation in cultured osteoblasts, and restore the activity of osteoblasts by repairing the damage caused by Runx2-deficiency^311^.

### Runx3

Runx3 is another member of the RUNT family^318^. Similar to Runx2, Runx3 also binds DNA in a heterodimer form with Cbfβ^319^ (Table 1). Runx3 is expressed in hematopoietic cells, skin appendages, and bone tissue^320,321^. Ablation of Runx3 controls early and late chondrocyte differentiation in Runx3-deficient embryos^322^ (Fig. 1). In addition, overexpression of Runx3 in chondrocytes leads to ectopic mineralization of the rib cage within mice, indicating that Runx3 plays a noteworthy role in cartilage development^323^. In contrast, Runx3-mutant mice developed severe congenital osteopenia, suggesting that Runx3 positively regulates osteoblast differentiation^324^ (Table 1).

In MSCs, BMP9 up-regulates endogenous Runx3 expression^325^. Runx3 overexpression increases BMP9-induced transcription factor expression, like Runx2 or DLX5, in osteoblasts, while Runx3 knockdown decreases the expression of these transcription factors^325^. In addition, Runx3 overexpression also leads to increased Smad1/5/8 phosphorylation induced by BMP, whereas Runx3 knockdown leads to a decrease^325^. As a result, Runx3 controls the induction of osteoblast differentiation by its interaction with BMP9^325^.

### Osterix

Osx has two effects on osteoblast differentiation, where it can both promote and inhibit osteoblast differentiation, depending on the situation (Fig. 4). Osx’s C-terminus comprises a DNA domain highly similar to the sequences found in Sp1, Sp3, and Sp4^326^. Osx also possesses a proline- and serine-rich domain that is capable of activating the expression of OCN and COL1A1^326^. Numerous MyF5-binding sequences and myoblast determination protein 2 (MyoD2)-binding sequences can be found in the proximal Osx promoter^327^. These basic helix-loop-helix structural transcription factors are essential for osteoblast differentiation, and Osx transcription may be controlled by these two sequences^327^. Zinc finger and BTB domain-containing 16, downstream target genes of Osx, act as late markers of osteoblast differentiation and thereby play a role in regulating osteogenesis in MSCs^328^. Fibrillin-2 and periosteal were also identified as candidate targets of Osx in osteoblast differentiation^329^. The transcription factor early B cell factor 1 (Ebf1) — known to regulate B cell, neuronal cell, and adipocyte differentiation — has also been shown to modulate osteoblast activity in a stage-dependent manner through its interaction with Osx, with Ebf1 promoting early osteoblast differentiation but hampering proper mature osteoblast function^330^.

While Runx2 is indispensable for osteoblast differentiation, certain mechanisms operate independently of Runx2^331,332^. For example, even though Runx2 is necessary for BMP-mediated Osx expression, Runx2 on its own is not enough to elicit Osx expression^136^. This suggests that DLX5, a transcription factor downstream of BMP, can also induce Osx expression^136^. The MAPK signaling pathway can also regulate Osx expression induced by BMP2 and IGF-1^333^. The transcription factor NFAT, which is particularly important to osteoclasts, can work with Osx in osteoblasts to speed up the process of differentiation^334^. Overexpression of NFATc1 has been shown to encourage the Osx-dependent activation of the COL1A1 promoter^335^. Through the activation of SOST and DKK1 expression, two significant inhibitors of the Wnt signaling pathway, Osx can suppress osteoblast activity^336^. By specifically binding to SOST gene promoter sequences, Osx can trigger SOST expression^337^. Thus, the Wnt signaling is inhibited, and osteoblast differentiation is disrupted. This intricate regulatory network is further underscored by the involvement of En1, which, due to Osx expression being delayed in En1 deficient mice, is expected that En1 is located upstream of Osx^338^. The manifestation of delayed skull ossification in mouse models with the En1 mutation indicates that En1 also plays a significant role in osteoblast differentiation^338^. In addition, ALP activity in osteoblast cells lacking En1 is reduced, with ALP being an essential enzyme in osteoblast differentiation^339^. OCN and BSP, two genes involved in the final stage of osteoblast differentiation, also have reduced expression when En1 is deleted^339^.

Recent investigations have delved into the impact of osterix stability on osteoblast differentiation, particularly in the context of E3 ligase complexes and ubiquitination processes. Within the evolutionarily conserved SCF ubiquitin ligase complex, F-box and WD repeat domain-containing 7 (FBW7) plays a crucial role as the substrate recognition element^340,341^. The phosphorylation of Osx at S73/77 by P38 facilitates its interaction with Fbw7, leading to subsequent Osx ubiquitination^341^. Furthermore, the depletion of Fbw7 in human mesenchymal stem cells and primary mouse calvarial cells resulted in an augmented osteogenic capacity^341^. Similarly, members of the Casitas B-lineage lymphoma family, Cbl-b and c-Cbl, have been identified as regulators that diminish the protein stability of Osx during BMP2-induced osteoblast differentiation through ubiquitin–proteasome-mediated degradation^342^. More recently, ubiquitin-specific protease 17 (USP17) has been found to increase Osx protein levels via modulating deubiquitylation, positively impacting osteoblast differentiation as evidenced by enhanced alkaline phosphatase (ALP) expression and activity^343^. These findings open a novel avenue for direct intervention and manipulation of Osx stability, providing a potential approach to enhance or inhibit osteoblast differentiation.

#### Co-activators of Osx

By binding to and activating CCACCC sites upstream of its promoter, Osx can control its own expression, forming a positive feedback mechanism^344^. BMP2 and Msh homeobox 2 (Msx2) trigger the expression of Osx in Runx2-mutant MSCs, and a mutation in Msx2 prevents the induction of Osx in Runx2-deficient MSCs (Table 1). This suggests that BMP2 independently controls Osx expression via Msx2^345^. Similarly, BMP2 and TGFβ-mediated Osx expression also require a novel factor, TGFβ-induced early gene 1 (Tieg1), to optimize Osx expression in osteoblasts^346–348^. Additionally, BMP2 directly regulates Osx expression by connecting with its proximal promoter, thus promoting osteoblast differentiation^346–348^.

**Fig. 5:**
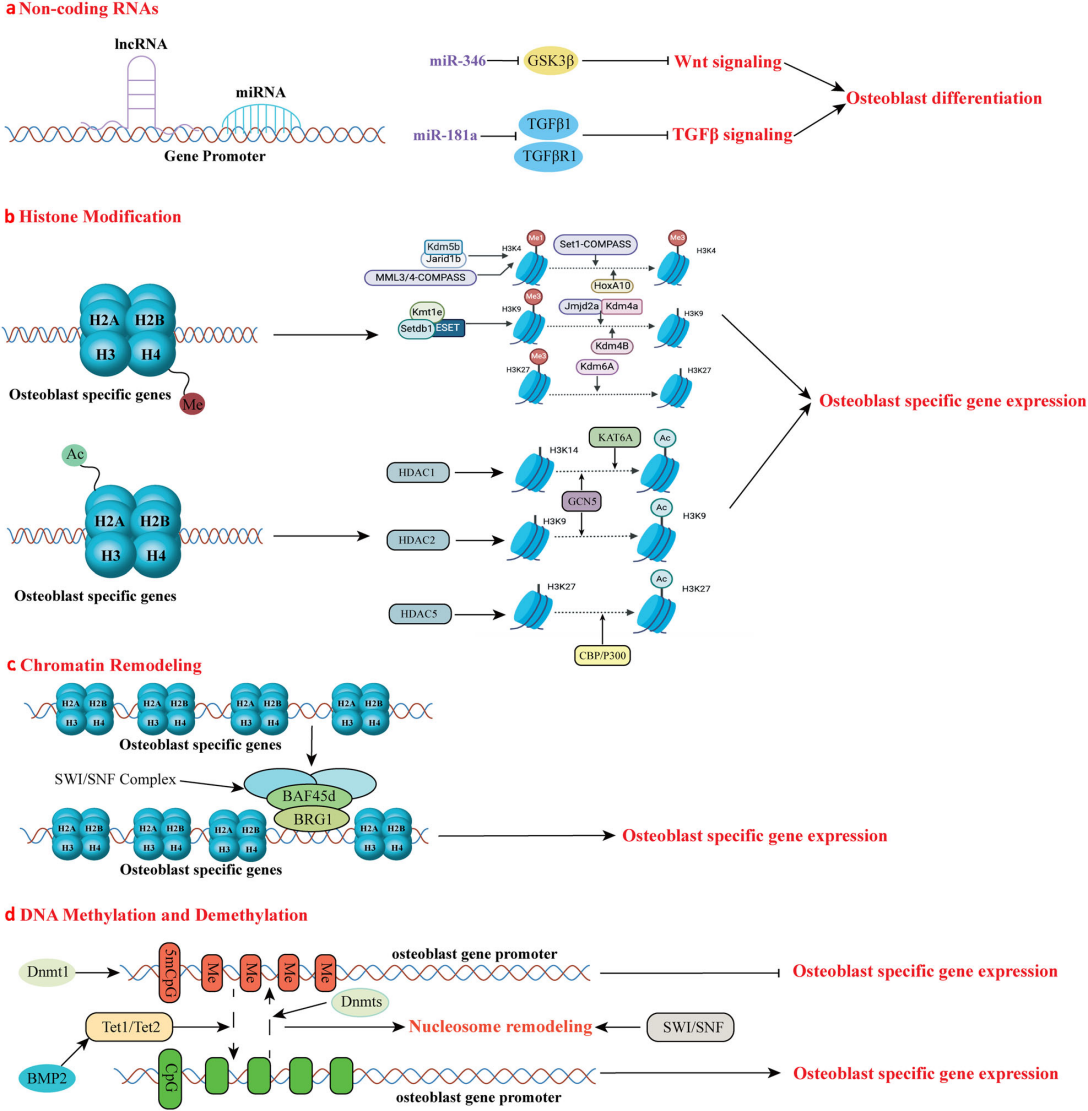
Epigenetic modification of transcriptional genes regulation in osteoblasts. **a** The non-coding RNAs have a regulatory effect on osteoblast differentiation; miRNA-346 can inhibit GSK3β to promote Wnt signaling activation, and miRNA-181a can inhibit TGFβ1 and TGFβR1 to promote TGFβ signaling activation to promote osteoblast differentiation. **b** The methylation and acetylation of histone H3 control the promoter of the transcriptional genes. The enrichment of H3K4me1, H3K9me3, and H3K27me3 leads to transcriptional silencing of gene promoters, while the enrichment of H3K4me3 promotes transcriptional activation. Demethylation of H3K9me3 and H3K27me3 also induces transcriptional activation of gene promoters. This group of proteins regulates the enrichment of different methylation groups and their methylation degree. Jarid1/Kdm5 can convert H3K4me3 and H3K4me2 to H3K4me1, while MLL3/4-COMPASS-like can catalyze the deposition of H3K4me1 on the enhancer elements. Set1-COMPASS and HoxA10 promote H3K4 methylation. Jmjd2a, Kdm4a and Kdm4B promote H3K9 demethylation. Kmt1e/Setdb1/ESET can mediate H3K9 trimethylation. Osteoblast differentiation is facilitated by the acetylation of H3K9, H3K14, and H3K27. HDAC1/2/5 promotes deacetylation of H3K9, H3K14, and H3K27, while GCN5 promotes H3K9 and H3K14 histone acetylation, KATA5 promotes H3K14 acetylation and CBP/P300 promotes H3K27 acetylation. **c** SWI/SNF regulates osteoblast differentiation through chromatin remodeling. BRG1 is one of the subunits of the SWI/SNF complex. Baf45d also belongs to SWI/SNF complex. When activated, it can regulate osteoblast early differentiation. SWI/SNF chromatin remodeling complex is required for Runx2-dependent initiation of skeletal gene expression. **d** The degree of DNA methylation affects the gene promoters. The Dnmts family, such as Dnmt1, can regulate DNA methylation while Tet1/2 can demethylate 5mCpG to promote Runx2 and Osx gene transcription, thereby promoting osteoblast differentiation. BMP2 can induce Tet1/2 activity to convert 5mCpG to 5hmCpG on Osx promoter.

Jumonji domain-containing 3 (Jmjd3) is also important in osteoblast differentiation and controls BSP and OCN expression through Runx2 and Osx^349^. When Jmjd3 was knocked out in mutant mouse models, Runx2 and Osx promoter activity decreased while H3K27me3 levels increased (Fig. 5)^349^. As a result, both DLX5 and Msx2 were unable to get close to these binding sites, preventing Runx2 and Osx from being activated^349^. Osteoblast differentiation was inhibited by increasing H3K27me3 occupancy of Runx2 and Osx promoter regions when Jmjd3 expression was silenced^349^. According to these findings, Jmjd3 positively regulates Osx function to exert transcriptional control over osteoblast lineage commitment^349^.

Panx3 acts as a highly expressed gap junction protein in bone cells that can regulate the initiation of osteoblast precursor cell differentiation. Panx3 can indirectly regulate the expression of Cx43, a key gene in osteoblast differentiation, by regulating the expression of Osx^350^. Panx3 acts as a hemichannel to release ATP, which can activate the PI3K/AKT signaling pathway^350^. Further opening of Panx3 ER Ca^2+^ channels leads to increased intracellular Ca^2+^ concentration, activation of CaM/NFAT signal transduction, and increased Osx expression^334^. Cx43 is the target gene of Osx, so Panx3 can regulate the differentiation process of osteoblasts by regulating the expression of Osx^351^.

#### Co-repressors of Osx

Osteoblast differentiation and Osx expression can be induced by inhibiting protein phosphatase 2Acα (PP2Acα), which can negatively regulate Osx expression^352^. Indeed, previous work has indicated that the silencing of PP2Acα expression led to an increased level of Osx expression, promoting the up-regulation of other bone-related genes, such as BSP and OCN^352^. Through MAPK and PKCδ signaling pathways, BMP2 and IGF-I can mediate Osx expression in MSCs. In addition, inhibition of PP2Acα significantly up-regulates Osx expression and its related genes, leading to the sustained differentiation of osteoblasts^352^.

NO66 is a protein containing a Jumonji C (JmjC) domain that directly interacts with Osx to inhibit Osx-mediated promoter activation^353^. Osteoblast differentiation and mineralization were accelerated when NO66 was knocked out of preosteoblasts, and Osx- targeted gene expression was significantly increased^353^. NO66 shows JmjC-subordinate histone demethylase movement, which is explicit for H3K4me and H3K36me (Fig. 5)^353^. Osx interacts with NO66 to regulate osteoblast-specific genes’ histone methylation status. This interaction between NO66 and Osx acts as histone demethylase to negatively regulate osteoblast gene expression^353^.

### ATF4

ATF4 is necessary for the timely onset of terminal osteoblast differentiation and controls osteoblast-specific gene expression (Table 1)^354,355^. The ATF/CREB protein family includes ATF4, the basic leucine zipper transcription factor^356^. Through the use of leucine domains, these proteins bind to DNA and transform into numerous dimers that coordinate the cellular regulation of signals from multiple pathways^357^ (Fig. 4). RSK2 and ATF4 direct type I collagen synthesis, an essential component of the bone matrix, following transcription^354^. Osteoblast-specific osteocalcin gene expression is triggered by the synergistic interaction between ATF4 and Runx2/Cbfa^358^. OCN is activated by the synergistic mutual effect between ATF4 and Runx2 at the 657 bp mOG2 promoter’s OSE1- and OSE2-binding sites, as determined by mutation analysis^358^.

Vimentin prevents osteoblast differentiation and OCN transcription, both of which are dependent on ATF4^359^. Vimentin binds to ATF4 in osteoblasts through the leucine zipper space, preventing osteoblast differentiation and ATF4-dependent OCN transcription^359^. This prevents ATF4 from binding to the OCN promoter’s cognate DNA, OSE1^359^. Additionally, the TGF-β signaling pathway regulates ATF4. Vimentin can be produced through the PI3K-Akt-mTOR signaling pathway, thereby inhibiting ATF4-dependent OCN transcription and osteoblast differentiation, even though it does not directly affect ATF4 expression^359^.

During bone formation, the dimerization of ATF4 with the C/EBP family is crucial. The heterodimerization of C/EBP with ATF4 is linked to one of the defining mechanisms of the delayed bone formation seen in C/EBP-deficient mice^360^. OCN transcription is triggered by these two proteins binding to the promoter of the OCN gene close to the ATF4-binding site^360^. C/EBP causes ATF4 to bind with Runx2, resulting in OCN expression^360^.

### SATB2

Matrix attachment region (MAR)-dependent transcription can be initiated by special AT-rich sequence-binding protein-2 (SATB2), which belongs to a distinct AT-rich family of binding proteins that bind to the nuclear MAR^361,362^. To regulate the chromatin structure, SATB2 collaborates with transfer-associated protein 2 (MAT2) and histone deacetylase 1 (HDAC1) (Table 1)^363^. In addition to directly activating or inhibiting DNA expression, SATB2 promotes DNA-binding protein activity by acting as a protein bracket^364^ (Fig. 4). SATB2 may play a significant role as a key node in the transcriptional regulatory network since it controls several important aspects of skeletal development^365^.

The BSP and OCN genes, which are essential for osteoblast differentiation, can be bound and controlled by SATB2. In addition, SATB2 prevented Hoxa2, a bone formation inhibitor, from being expressed (Fig. 1)^366,367^. Through physical interaction, SATB2 strengthened ATF4’s and Runx2’s ability to bind to similar DNA recognition elements^368^. By binding to the SATB2 promoter, Osx can directly control the expression of SATB2^369^. BMP4 may regulate upstream of SATB2, as evidenced by the fact that SATB2 was up-regulated in the mandible of mice overexpressing BMP4 but down-regulated in the mandible of mice lacking BMP4^370^. SATB2 expression was also increased when Osx was overexpressed^371^. Mechanistically, Osx can bind directly to the 130-bp GC-rich SATB2 promoter binding site at the 368 proximal end, activating SATB2 expression^371^. As a result, Osx is an upstream regulator of SATB2, with SATB2 transcription occurring after BMP signaling^371^. SATB2 expression was inversely correlated with TNF-α content^369^. The mesenchymal cell line C2C12 osteoblasts can be constrained by TNF-α, reducing the expression of SATB2 induced by BMP^369^. Therefore, TNF-α is a negative regulator of osteoblast differentiation and can inhibit the differentiation of osteoblasts by inhibiting the expression of SATB2^369^.

### TAZ/YAP

A transcriptional regulator known as TAZ has a domain that binds to PDZ (Table 1). YAP is a significant downstream effector in the Hippo signaling pathway (Table 1)^372^. Bone formation is one area in which YAP/TAZ plays a significant role. TAZ can interact with Runx2 and PPAR, promoting Runx2 transcription and inhibiting PPAR, thereby preventing adipocyte generation and promoting osteoblast differentiation^373^. The transcriptional regulator LEF is crucial for osteoblast-specific gene expression within the Wnt/β-catenin signaling pathway^374^. TAZ combines with LEF to create a complex that promotes Wnt3a signaling and regulates Runx2 function^375^. By binding to Runx2, YAP can stop Runx2 activation. The Runx2-YAP complex can be separated by hindering Src/Yes kinase or obstructing tyrosine phosphorylation of YAP^247^. Because the interaction between YAP and Runx2 is facilitated by tyrosine phosphorylation of endogenous YAP, Runx2 can recruit phosphorylated YAP to the OCN promoter and the subnuclear site of Runx2, inhibiting OCN promoter activity^247^. Snail (SNAI1) and Slug (SNAI2) form a binary complex with YAP/TAZ that regulates the protein level of YAP/TAZ (Fig. 3)^376^. This protects YAP/TAZ from proteolytic hydrolysis and controls the expression of YAP/TAZ-targeted genes, positively regulating osteoblast differentiation^376^.

### Cbfβ

Core binding factors (Cbfs) are heterodimeric transcription factors with two subunits: Cbfα (Runx proteins) and Cbfβ. The Cbfβ subunit functions as a non-DNA binding factor that can bind to Runx (Cbfα) and encourage gene expression (Table 1). Numerous developmental processes, including the formation of the skeletal system, are influenced by the Runx/Cbfβ complex (Fig. 4). Cbfβ can not only bind to the Runx protein, but also able to interact with other osteoblast differentiation-related transcription factors like ATF4 and Osx^377^. This interaction has the potential to significantly up-regulate the transcriptional expression of ATF4 and Osx^377^. Cbfβ deficiency can lead to the down-regulation of chondrocyte differentiation-related gene expression, impairing chondrogenesis^378^. Cbfβ-mediated chondrocyte maturation is also important for trabecular bone morphogenesis^378^. For instance, Cbfβ-deficient mice suffer from bone defects and die during embryonic development as a result of a lack of hematopoiesis and subsequent hemorrhaging^379,380^. Furthermore, not only does down-regulation of the Cbfβ gene delay the development of the sternum, but it also impacts the growth of numerous bones, such as the skull, tibia, femur, spine, and ribs^381^.

Through the formation of a binding complex, Cbfβ stabilizes Runx2 in osteoblasts and promotes Runx2’s regulation of osteoblast-specific gene expression (Fig. 4). By inhibiting the ability of WW domain-containing E3 ubiquitin protein ligase 1 (WWP1) to bind to Runx2, the Runx2/Cbfβ complex can prevent Runx2 from being ubiquitinated and subsequently degraded^379^. Additionally, the disruption of the Runx2/Cbfβ complex via small osteoblast vesicles containing micro-RNA that suppresses the expression of Cbfβ was shown to inhibit the transformation of pre-osteoblast cells to mature osteoblast cells^382^. The Runx/Cbfβ complex can directly increase transcriptional Ihh expression and promote osteoblast differentiation by combining with the Runx binding site in the Ihh promoter, controlling Runx2 acetylation, phosphorylation, and isomerization, as well as other posttranslational modifications^380^. The maturation of osteoblasts and their transformation into osteocytes are slowed down when Runx2 and Cbfβ are overexpressed in osteoblasts^379^.

## Epigenetic modification of transcriptional gene regulation

The study of epigenetic modification includes DNA methylation, acetylation, and ubiquitination, as well as the chromatin regulatory proteins that control changes in gene expression. The epigenetic regulation during osteoblast differentiation has progressed significantly in recent years. Epigenetic inheritance plays a crucial role in gene expression by regulating chromatin structure to dynamically control gene expression via permitting or declining access of transcriptional machinery to chromatin. Non-coding RNAs, DNA modifications, chromatin remodeling, and the dynamic epigenetic regulatory process are all essential for osteoblast development. The methylation and acetylation of DNA and histones have received the most attention (Fig. 5; Table 3).

**Table 3:** Epigenetic proteins regulating osteoblast differentiation.

Enzyme	Name	Function	Reference
Histone methylases	EZH2	Catalyzes the trimethylation of H3K27 and inhibits Runx2 and OCN expression.	^392^
	Kmt1e/Setdb1/ESET complex	Mediates H3K9 trimethylation.	^394^
	Set1a/b-COMPASS	Transits H3K4me1 to H3K4me3.	^400^
Histone demethylases	KDM6B	Removes H3K9me3 and H3K27me3 methyl group.	^390,391^
	KDM4B	Removes H3K9me3 and H3K27me3 methyl group.	^390,391^
	Jarid/Kdm5	Converts H3K4me3 and H3K4me2 to H3K4me1.	^390^
Histone acetylases	GCN5	Regulates acetylation of histones at residues K9 and K14.	^410^
	KAT6A	Responsible for H3K9 and H3K14 acetylation.	^411^
	CBP/P300	Mediates H3K27 acetylation and the activation of Foxo1.	^412^
Histone deacetylases	SIRT1	Enhances Runx2 trans-activation activity.	^301^
	HDAC1	Negatively regulates osteoblast generation by reducing Osx and OCN gene promoters’ H3 and H4 acetyl labeling.	^403^
	HDAC2	Expressed in bone tissue and activates Akt expression, which inhibits FoxO1 transcription and leads to RANKL-induced osteoblast generation.	^403^
	HDAC5	Binds with MEF2C to inhibit SOST expression and reduce H3K27 acetylation levels at the SOST gene site to promote Wnt/β-catenin signaling.	^408^
DNA methylases	Dnmt3a	Inhibits osteoblast differentiation via regulating Wnt/β-catenin signaling and Runx2, Ocn activity.	^422^
DNA demethylases	TET1/2	Converts 5mCpG to 5hmCpG.	^423–425^
Chromatin remodeling	Baf45a/ Baf45d	Promote the activation of genes crucial for osteoblast maturation and mineralization	^415,419,420^

### Non-coding RNAs

MicroRNAs (miRNAs) are noncoding RNA molecules that exert a negative regulatory effect on target gene expression through mRNA degradation or translational repression^383^. Past studies have found that miRNAs play an important role in osteoblast differentiation^384,385^. A recent study found that miR-346 can directly bind to the 3’-untranslated region (UTR) of GSK-3β mRNA, and miR-346 can promote osteogenic differentiation by repressing GSK-3β and activating the Wnt/β-catenin pathway^41^ (Fig. 5a). Another study found that miR-181a is highly upregulated during BMP-induced osteoblastic differentiation. They demonstrated that miR-181a targets the negative regulator of osteoblastic differentiation Tgfbi (Tgf-beta induced) and TβR-I/Alk5 (TGF-β type I receptor), thereby promoting osteoblastic differentiation via repression of TGF-β signaling molecules^386^ (Fig. 5a). Therefore, the miRNAs are important in osteoblast differentiation via regulating osteoblast differentiation-specific genes^386^.

### Histone methylation

Chromatin reorganization and gene transcription are both regulated by methylation of the histone. Lysine (K) methylation of H3K9^387^ and H3K27^388^ is associated with transcriptionally silenced regions. By removing H3K9me3 and H3K27me3, the histone demethylases KDM4B and KDM6B are important in MSC differentiation (Fig. 5b; Table 3)^389^. Osteogenic differentiation was significantly reduced when lysine demethylase 4B (KDM4B) or lysine demethylase 6B (KDM6B) were depleted^390,391^. The epigenetic switch for human MSC differentiation can be regulated by the mutual and concerted effect of enhancer of zeste homolog 2 (EZH2) and lysine demethylase 6 A (KDM6A)^391^. EZH2, the catalytic subunit of Polycomb repressive complex 2 (PRC2), plays a crucial role in gene repression by catalyzing the trimethylation of H3K27^392^. H3K27me3 is specifically trimethylated by EZH2, whereas the methyl group is removed by KDM6A^392^. The histone H3K27 methyltransferase EZH2 negatively regulates osteogenesis, and the expression of Runx2 and OCN is inhibited in the presence of EZH2 during osteogenic differentiation because H3K27me3 is dramatically increased on Runx2 and OCN transcription start sites (TSS) (Fig. 5b; Table 3)^393^. Cyclin-dependent kinase 1 (CDK1) can phosphorylate EZH2 at Thr 487. This phosphorylation event not only hinders the binding of EZH2 to its targets on the promoter but also inhibits the histone methyltransferase (HMTase) activity of EZH2 by disrupting the PRC2 complex^392^. The removal of repression of the putative EZH2-target genes, facilitated by the phosphorylation of EZH2 at Thr 487 by CDK1, results in the promotion of MSC osteogenic differentiation^392^. Kmt1e/Setdb1/ESET can mediate H3K9 trimethylation^394^.

Methylation of H3K4 is associated with transcriptionally active regions^395^. Trxg-mediated activity encompasses monomethylation, dimethylation, and trimethylation of lysine-4 residues of histone H3 (H3K4me1, H3K4me2, and H3K4me3)^396^. The Jarid1/Kdm5 family (Jarid1a, b, c, and d) is capable of converting H3K4me3 and H3K4me2 to H3K4me1 (Fig. 5b; Table 3)^390^. H3K4me3 is commonly found in transcriptionally active chromatin (euchromatin), primarily concentrated around the transcription initiation site (TSS) of promoters^394^. The global genomic deposition of H3K4me3 in most mammalian cells is regulated by Set1-COMPASS, thereby closely linking its function to the transcriptional activation of a large number of genes^397^. MLL3/4-COMPASS-like complexes can catalyze the deposition of H3K4me1 on the enhancer elements of mammalian cells^398,399^. The reduction of MLL3/4 results in the transcriptional activation of specific MLL3/4 target genes. This happens because both Set1a/b-COMPASS and MLL1-COMPASS can reach these genes, aiding in the shift of associated transcription from H3K4me1 to H3K4me3^400^. Enrichment of H3K4me1 typically results in the inhibition of osteoblast differentiation. Furthermore, the accumulation of H3K4me1 suppresses the expression of key transcription factors in osteoblasts, such as Runx2 and Osx (Fig. 5b; Table 3)^401,402^. Methylation of H3K4, particularly the enrichment of H3K4me3, facilitates osteoblast differentiation. In this context, crucial transcription factors of osteoblasts like Runx2 and Osx become transcriptionally active (Fig. 5b; Table 3)^402^.

### Histone acetylation

Histone acetylation is regulated by histone deacetylase (HDAC) and histone acetyltransferases, which affect chromatin conformation and regulate gene transcription. Acetylation of a histone typically facilitates gene transcription by allowing transcription factors and RNA polymerase II access to the DNA via relaxing the chromatin structure. In this manner, histone acetylation encourages gene transcription, while histone deacetylation causes chromatin to become compacted and prevents gene expression^403^. During osteoblast differentiation, H3K9Ac is linked to genes that are activated, and H3K9me2 is linked to genes that are silenced (Fig. 5b; Table 3)^394^. Sirtuin 1 (SIRT1) is a Class III histone deacetylase^301^. SIRT1 activity can promote osteoblast differentiation^301^. By interacting with Runx2, SIRT1 can enhance its trans-activation activity^301^. As Osx is one of Runx2’s downstream targets, SIRT1 can trigger Runx2’s activation and promote Osx expression, which in turn targets osteoblast-specific gene expression and promotes osteoblast differentiation^301^.

HDACs, or histone deacetylases, are a class of enzymes responsible for removing acetyl groups from both histone and non-histone proteins. This action facilitates the tightening of DNA wrapping around histones^404,405^. HDAC1 exerts a negative regulatory effect on osteoblast generation by reducing the acetylation levels of histones H3 and H4 on the promoters of key osteoblast-related genes such as Osx and OCN (Fig. 5b; Table 3)^403^. HDAC1 can bind to the promoter of Runx2, thereby abolishing its transcriptional activity and consequently exerting its inhibitory effect on osteogenesis in the absence of osteogenic signals^403^. During osteogenesis, rapidly proliferating osteogenic progenitors exit the cell cycle to differentiate into functional osteoblasts. The subsequent arrest of the cell cycle is crucial for osteoblast differentiation^406^. HDAC1 interacts with promoters of cell cycle inhibitors such as P27, impeding the progression of the osteogenic lineage^407^. HDAC2 is broadly expressed in bone tissue and enhances Akt expression. This upregulation of Akt inhibits FoxO1 transcription, consequently promoting RANKL-induced osteoblast generation^403^. HDAC5 binds to MEF2C, leading to the inhibition of SOST expression. This action reduces H3K27 acetylation levels at the SOST gene site, thereby promoting Wnt/β-catenin signaling^408^.

General control non-repressed protein5 (GCN5) is a histone acetyltransferase (HAT) that plays a crucial role in normal embryogenesis. Its function involves gene transcriptional activation and the regulation of a specific chromatin program characterized by histone acetylation^409^. Gcn5 is associated with the acetylation of histones at residues K9 and K14, which are responsible for gene activation^410^. Previous work has studied the epigenetic regulation mechanism of Gcn5 on osteoblast differentiation in an inflammatory environment^410^. During inflammation, Gcn5 expression is reduced, which leads to Wnt/β-catenin development and impingement of PDLSC osteogenesis. Gcn5 regulates osteoblast differentiation by depositing acetyl markers on histone H3 at lysine 9 and lysine 14 (H3K9 and H3K14) in the DKK1 promoter region (Fig. 5b; Table 3)^410^. DKK1 inhibits Wnt/β-catenin signaling by upregulating DKK1 in periodontal ligament stem cells (PDLSCs), thereby promoting osteogenesis with support from Gcn5^410^. KAT6A, an HAT belonging to the MYST family, is responsible for acetylating histone H3 at lysine 9 and lysine 14 (H3K9 and H3K14) (Fig. 5b; Table 3)^411^. In the context of cellular senescence, the levels of KAT6A protein and Nrf2/ARE signaling decrease as a result of stem cell dysfunction. Overexpression of KAT6A leads to the promotion of the Nrf2/ARE pathway through altered histone H3 acetylation patterns, thereby restoring cellular stemness^411^. Besides, CREB-binding protein (CBP) (CREBBP) and p300 (EP300) are multifunctional HATs with broad homology (Fig. 5b; Table 3)^412^. One study reported that conditional knockout of CBP or p300 in osteoblasts inhibited bone formation, resulting in reduced bone mass and strength^412^. Mechanistically, CBP/p300 HAT regulates the expression of osteogenic genes partly by activating β-catenin transcriptionally and suppressing Stat1. The acetylation of histone H3K27 and the transcription factor Foxo1 have been demonstrated to play roles in the CBP/p300 HAT-mediated transcriptional regulation of β-catenin and STAT1, respectively^412^(Fig. 5b; Table 3). These findings indicate that the acetyltransferase CBP/p300 could be a key regulator in promoting osteoblast differentiation^412^.

### Chromatin remodeling

The switch/sucrose non-fermenting (SWI/SNF) chromatin remodeling complex comprises multiple subunits engaged in diverse cellular processes, such as gene regulation, cell cycle modulation, development, and differentiation^413^. The mammalian SWI/SNF complex contains a catalytic subunit, BRG1 or BRM, which includes ATPase activity^414,415^. Mutations in the ATPase domain of BRG1 or BRM disrupt ATP binding, leading to the formation of inactive SWI/SNF complexes^414,416,417^. When mutant BRG1 or BRM proteins were expressed in NIH3T3 cells, it hindered the cells’ capability to activate endogenous stress response genes and to undergo differentiation into muscle or fat cells in the presence of arsenite^414,416^. Mutant BRG1 protein in NIH3T3 cell lines has been shown to inhibit BMP2-induced osteoblast lineage differentiation^418^. SWI/SNF is required for BMP2-induced alkaline phosphatase (APase) expression, an early marker reflecting Runx2’s control of osteoblast differentiation^418^. BRG1 is expressed in developing bone cells in mouse embryos and in vitro osteoblasts^418^. These results suggested that MP2-mediated osteogenesis requires Runx2 and suggest that the SWI/SNF chromatin remodeling complex is required for BMP2-induced, Runx2-dependent initiation of skeletal gene expression (Fig. 5c; Table 3)^418^.

Baf45a and Baf45d, two homologs involved in chromatin remodeling, have been demonstrated to be expressed in osteoblasts during maturation (Fig. 5c; Table 3). BAF45A is associated with the polybromo-associated BAF (PBAF) complex, while the BAF45D subunit belongs to the polymorphic canonical BRG1-associated factor (cBAF) complex^419,420^. Both BAF45A and BAF45D belong to the subunits of SWI/SNF complex^415^. During BMSCs differentiation, chromatin immunoprecipitation sequencing (ChIP-seq) revealed elevated histone H3K9 and H3K27 acetylation modifications in the promoter regions of Baf45a and Baf45d, accompanied by enhanced binding of RUNX2^420^. The overexpression of Baf45a in osteoblasts triggered the activation of genes crucial for osteoblast maturation and mineralization^420^. Conversely, Baf45a knockdown in odontoblasts led to significant changes in the expression of genes associated with proliferation, apoptosis, DNA repair, and a modest reduction in dentinogenic marker gene expression^420^. Additionally, Baf45a knockout osteoblasts exhibited a noticeable decrease in chromatin accessibility for osteoblast and odontoblast-specific genes, including the transcription factor Atf4^420^. These findings suggest that BAF45A-dependent chromatin remodeling, facilitated through PBAF-RUNX2 crosstalk, plays a pivotal role in the transcriptional activation essential for the early differentiation and matrix maturation of mineralized tissues^420^.

### DNA methylation and demethylation

DNA methylation entails the epigenetic process of adding a methyl group to the C5 position of cytosine, resulting in the formation of 5-methylcytosine^421^. DNA methylation is mainly regulated by a family of DNA methyltransferases (Dnmts), which are found to be important in osteoblast differentiation^421^. A recent study found that Dnmt3a may inhibit osteoblast differentiation via regulating Wnt/β-catenin signaling and Runx2, Ocn activity (Fig. 5d; Table 3)^422^. Demethylation of the promoter’s CG region is linked to the ten-eleven translocases (TET) protein, which may alter chromatin status and encourage osteoblast differentiation^422^. The TET protein forms a complex with Runx2 to mediate the demethylation of the Runx2 promoter and subsequently promote Runx2 expression^423^ (Fig. 5d; Table 3). By recruiting the TET protein into the Runx2 promoter region, the resulting hypomethylation of DNA can provide a more accessible chromatin environment for RNA Pol II, promoting gene expression^423^. These findings demonstrate that TET enzymes function to regulate Runx2 activity and maintain skeletal homeostasis^423^. During BMP2-induced osteoblast differentiation, DNA demethylation of the Osx promoter is mediated by a Tet1/Tet2-containing complex that converts 5mCpG to 5hmCpG^424^. A recent study also found that Tet1 recruits Tet2 to mediate 5mCpG demethylation and gene transcription during BM-MSC differentiation^425^ (Fig. 5d; Table 3).

The expression of osteoblast genes may also be controlled via the methylation mechanism of Vitamin C (ascorbic acid, AA), a well-known regulator of bone and cartilage metabolism. A recent study confirmed that AA exerts differential effects on osteoblast differentiation, with AA increasing levels of 5-hydroxymethylcytosine (5-hmC) and inducing DNA demethylation via the ten-eleven translocases (TETs)^426^. Prolyl hydroxylase domain-containing protein 2 (PHD2), recognized as a mediator of AA’s effects in these tissues, belongs to the same enzyme family as the TETs^426^. Blocking PHD2 resulted in decreased 5-hmC levels in promoters of genes associated with chondrocyte differentiation^426^. Knockdown of Phd2 in chondrocytes also led to a reduction in global 5-hmC levels, indicating that PHD2 might directly contribute to increases in 5-hmC in genes related to chondrocyte and osteoblast functions^426^.

## Summary and future perspectives

Embryonic bone formation and the dynamic remodeling of adult bone are processes by which mesenchymal cells of the osteoblast lineage transform into mature osteocytes in mineralized connective tissue. The intricate interaction of signaling proteins, transcription factors, and co-regulatory proteins supports these processes^279^. Numerous transcription factors and signaling pathways that occupy important positions in osteoblast differentiation have been elucidated over the previous decades thanks to advances in biomolecular techniques. Numerous studies and data support the hypothesis that Runx family proteins and the Wnt signaling pathway regulate osteoblast differentiation. Different signaling pathways contain a series of molecular interactions, which provide potential targets for clinical drug therapy. Many signaling pathways have been implicated in osteoblast differentiation, such as Wnt, Notch, and Hippo, making them attractive targets for the research of drugs to treat bone diseases. With the progress of technology, the factors that participate in osteoblast differentiation have been gradually discovered. However, the regulation of osteoblast differentiation is a large and complex network that involves not only intracellular proteins and genetic interactions but also communication between cells. As such, osteoblast differentiation research faces many unanswered questions and challenges that still need to be overcome, such as:

1. The mechanisms behind the temporal and spatial regulation of many essential signaling and transcription factors (such as BMP or Runx2) in osteoblast differentiation during different stages remain unclear.
2. Some signaling pathways have bi-directional effects on osteoblast differentiation, like the Notch signaling pathway and TGFβ signaling pathway. How this bidirectional effect regulates osteoblast differentiation and under what circumstances it promotes or inhibits osteoblast differentiation still needs to be determined.
3. In addition to the effect of signaling pathways on osteoblast differentiation, cell−cell interactions, such as osteoblast and osteoclast interaction, and the genetic regulation that controls the mutual transformation between osteoblasts, chondrocytes, and adipocytes, still requires further investigation.
4. With developments in molecular biology and genetics, epigenetic regulation has become a major source of intrigue within the study of osteoblast differentiation. A major focus has been afforded to how histones are modified, the regulation of chromatin structure, and how small RNAs function in regulating gene expression. The interaction and coordination of these regulatory processes are also worth studying, such as how chromatin is “turned on” to expose genes, enabling transcription factors to control the expression of pivotal genes. Epigenetic changes at various destinations will produce different outcomes, and the epigenetic control at the various phases of osteoblast differentiation should be examined.
5. Bioinformatic technology can also be used to study the transcriptional regulation of osteoblast differentiation. For example, bioinformatic skills such as ATAC-seq can be used to study how enhancers regulate gene expression^427^.
6. In the regulation of osteoblast signaling, many molecules are involved in skeleton-related diseases. Therefore, many proteins in the Wnt signaling pathway are promising targets for clinical therapy. Determining which targets can be used and how to design efficacious pharmaceutical drugs that can treat these skeleton-related diseases with minimal side effects is also a current challenge in this area of clinical research.
7. Osteoporosis has been studied in different animal models; yet these models do not satisfactorily resemble the features of the human disease phenotype^428^. Although experimental modeling of human bone diseases represents a breakthrough by allowing greater insight into the cellular and molecular mechanisms involved in bone pathology, there are shortcomings in using MSC models. Limited availability of MSCs in patients, extreme heterogeneity, limited proliferative capacity, and loss of function are the most common limitations when using MSCS in vitro^429^. These limitations on clinical treatment are also issues that need to be addressed.
8. As our comprehension of cellular dynamics during bone remodeling advances, a plethora of therapeutic targets has emerged from preclinical studies, encompassing membrane expression, cellular crosstalk, and gene regulation. These targets show promising therapeutic effects, offering diverse options for drug development in treating bone-related diseases. Additionally, drugs utilizing carrier transport mechanisms have been devised for this purpose^430^. Nonetheless, our current understanding of bone biology and its interplay with other systems remains incomplete. The potential impacts on the entire bone remodeling process and other physiological systems, rather than solely focusing on the bone microenvironment or individual cell−cell interactions, represent a fertile area for investigation. Further research is needed to identify more precise targets in order to optimize therapeutic outcomes.
9. Bone tissue has numerous vital functions and is closely tied to many important tissues and organs’ biochemical, homeostatic, and pathophysiological functions. Because of this critical link, many diseases are also associated with musculoskeletal diseases, such as obesity, cancer, bone metastases, and kidney diseases. Therefore, interdisciplinary research on musculoskeletal diseases, including obesity, cancer, kidney, and other diseases, may provide new insights into bone-related diseases and vice versa.

Currently, the drugs used in the clinical treatment of osteoporosis-related diseases focus on inhibiting osteoclastic bone resorption. In contrast, the drugs targeted at osteoblasts are mainly sclerostin inhibitors that maintain osteoblasts’ function by inhibiting sclerostin’s inhibition on the Wnt signaling pathway^431^. However, a more recently developed inhibitor of Fap has also been found to be an effective drug for osteoporosis^432^. The classical signaling pathway is an appealing target for clinical drug development. Wnt, BMP, and other classical signaling pathways have many known key signaling factors, such as β-catenin and Smad proteins. These factors can also be used as clinical targets to make corresponding activators or inhibitors. Researching compelling small-molecule activators or inhibitors to manage the pathway might emphatically affect the treatment of bone-related diseases like osteoporosis. Therefore, more therapeutic targets and new pharmaceutical research strategies can be developed to treat a diverse range of disease pathologies by studying the regulation of signaling pathways and transcription factors during the differentiation of osteoblasts.
